# Reduced thalamic excitation to motor cortical pyramidal tract neurons in parkinsonism

**DOI:** 10.1126/sciadv.adg3038

**Published:** 2023-08-23

**Authors:** Liqiang Chen, Samuel Daniels, Rachel Dvorak, Hong-Yuan Chu

**Affiliations:** ^1^ Department of Neurodegenerative Science, Van Andel Institute, Grand Rapids, MI 49503 USA.; ^2^ Aligning Science Across Parkinson’s (ASAP) Collaborative Research Network, Chevy Chase, MD, 20815.

## Abstract

Degeneration of midbrain dopaminergic (DA) neurons alters the connectivity and functionality of the basal ganglia-thalamocortical circuits in Parkinson’s disease (PD). Particularly, the aberrant outputs of the primary motor cortex (M1) contribute to parkinsonian motor deficits. However, cortical adaptations at cellular and synaptic levels in parkinsonism remain poorly understood. Using multidisciplinary approaches, we found that DA degeneration induces cell subtype– and input-specific reduction of thalamic excitation to M1 pyramidal tract (PT) neurons. At molecular level, we identified that *N*-methyl-d-aspartate (NMDA) receptors play a key role in mediating the reduced thalamocortical excitation to PT neurons. At circuit level, we showed that the reduced thalamocortical transmission in parkinsonian mice can be rescued by chemogenetically suppressing basal ganglia outputs. Together, our data suggest that cell subtype– and synapse-specific adaptations in M1 contribute to altered cortical outputs in parkinsonism and are important aspects of PD pathophysiology.

## Teaser

Cortical neuroplasticity contributes to parkinsonian pathophysiology.

## INTRODUCTION

Degeneration of dopaminergic (DA) neurons in the substantia nigra pars compacta (SNc) alters the magnitude and temporal pattern of basal ganglia (BG) outputs to downstream motor regions and has been causally linked to the motor deficits in Parkinson’s disease (PD) (*1*–*3*). The substantia nigra pars reticulata (SNr) is the major BG output structure in rodents. SNr neurons synthesize and release γ-aminobutyric acid (GABA) as neurotransmitter and send inhibitory projections to the motor region of the thalamus (mTh), including the ventromedial (VM) and ventral anterior-lateral (VAL) thalamus (*4*–*7*). The mTh transmits motor selection and execution information to cortical regions for proper motor control (*8*–*11*). Thus, in PD the thalamocortical network occupies a critical position in mediating motor symptoms, particularly the disruption of voluntary movements (*12*–*16*).

The primary motor cortex (M1) plays a key role in sensorimotor integration and proper control of motor behaviors (*17*–*19*). Its involvement into pathophysiology of parkinsonian motor symptoms was proposed by the rate model of BG-thalamocortical network and further supported by recent experimental evidence (*1*, *12*–*14*, *20*–*22*). However, considering the heterogeneity and complexity of cortical cell subtypes and their associated synaptic connections, our understanding of M1 circuit dysfunction in PD remains limited. For example, how do BG dysfunction and/or DA neurodegeneration affect the structural and functional connectivity of M1 circuits? How does M1 circuit dysfunction at macroscopic and microscopic levels contribute to PD symptoms? Answers to these questions are needed to advance our understanding of circuit pathophysiology in PD.

Research suggests that M1 circuits exhibit specific alterations in parkinsonism. Loss of SNc DA neurons alters neuronal firing and synaptic connectivity of M1 pyramidal neurons (*12*, *23*–*25*). Particularly, DA depletion selectively affects the activity of pyramidal tract (PT) neurons, but not the intratalencephalic (IT) neurons, in the layer V of M1 (*12*, *14*, *21*, *26*). These structural and functional changes perhaps contribute to the rewired cortical motor representation and impaired forelimb function in parkinsonian state (*27*–*29*). Building on these observations, we hypothesized that degeneration of SNc DA neurons induces cell subtype– and input-specific adaptations in M1 circuits. Our results largely agree with our predictions and provide a detailed description that contributes to mechanistic understanding of M1 circuitry adaptations in parkinsonism.

## RESULTS

Mice received either 6-hydroxydopamine (6-OHDA) injection into the medial forebrain bundle (MFB) to induce the degeneration of nigrostriatal pathway and SNc DA neurons (“6-OHDA mice” herein) or vehicle injection into the MFB as controls (“controls” herein). Physiological and anatomical studies were conducted between 3 and 5 weeks postinjections.

**Fig. 1.**
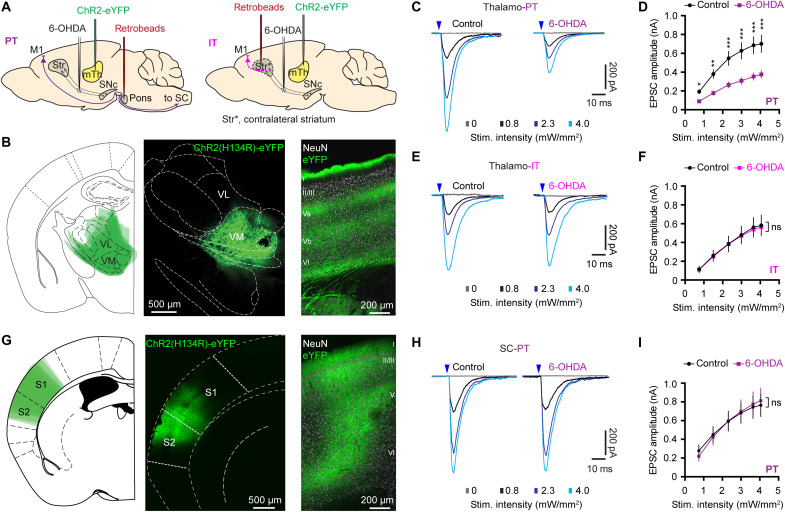
SNc DA degeneration induces cell subtype– and input-specific alterations in M1. (**A**) Overall strategies to label projection defined M1 pyramidal neurons and optogentically activate thalamocortical transmission arising from the mTh. (**B**) Overlaid image showing the averaged AAV injection sites in the motor thalamus (left), as well as representative confocal images showing an example of viral injection site in the motor thalamus (middle) and eYFP-expressing thalamic axon terminals in the M1 (right). eYFP-labeled terminals concentrate in the layers I and V and are consistent with the overall pattern of motor thalamic innervation of M1 in rodents. (**C** and **D**) Representative traces of optogenetically evoked EPSCs across different stimulation intensities in PT neurons from controls and 6-OHDA mice (C) and the summarized results (D). *P* < 0.0001 between groups, mixed effects model followed by Sidak’s tests. *P < 0.05, **P < 0.01, ***P < 0.001 relative to controls. Control = 62 cells per seven mice; 6-OHDA = 73 cells per eight mice. (**E** and **F**) Representative traces of optogenetically evoked EPSCs across different stimulation intensities in IT neurons from both controls and 6-OHDA mice (E) and the summarized results (F). ns, not significant between groups, mixed effects model. Twenty-six cells per three mice for each group. (**G**) AAV-infected cortical regions were overlaid to show the averaged AAV infection region across animals (left) and representative confocal images showing AAV9-ChR2(H134R)-eYFP infusion site in the sensory cortical (SC) areas (middle) and eYFP-expressing cortical axon terminals in the M1 (right). (**H** and **I**) Representative traces of optogenetically evoked EPSCs arising from the SC across different stimulation intensities in M1 PT neurons from both controls and 6-OHDA mice (H) and the summarized results (I). Control = 20 cells per three mice, 6-OHDA = 26 cells per three mice. Mixed effects model, not significant between groups. Arrowheads in (C), (E), and (H) indicate the timing of the optical stimulation.

To interrogate synaptic properties of thalamocortical inputs to projection-defined subtypes of cortical pyramidal neurons in controls and 6-OHDA mice, we stereotaxically injected adeno-associated viral (AAV) vectors encoding channelrhodopsin 2 [AAV9-hSyn-ChR2(H134R)-eYFP] into the mTh, centering at the VM thalamus (Fig. 1, A and B). Retrobeads were injected into the ipsilateral pontine nuclei and contralateral striatum to label PT and IT neurons, respectively (Fig. 1A). Three weeks postinjection, intense enhanced yellow fluorescent protein (eYFP)–labeled fibers could be observed, particularly in layers I and V of M1 (Fig. 1B) (*8*, *30*). Synaptic strength of thalamic inputs to retrogradely labeled neurons was measured as the amplitude of optogenetically evoked excitatory postsynaptic currents (oEPSCs) at a holding potential of −80 mV, which were mainly mediated by α-amino-3-hydroxy-5-methyl-4-isoxazolepropionic acid (AMPA) and *N*-methyl-d-aspartate receptors (NMDARs) (fig. S1).

### SNc DA degeneration induces cell subtype– and input-specific alterations in M1

Whole-cell voltage-clamp recordings were conducted in the presence of tetrodotoxin (TTX; 1 μM) and 4-aminopyridine (4-AP; 100 μM) to prevent the engagement of polysynaptic responses and facilitate optogenetically evoked glutamate release from thalamic axon terminals (*8*, *31*, *32*). The amplitude of thalamic oEPSCs in PT neurons (thalamo-PT) from the ipsilateral hemisphere with lesion was greatly reduced in 6-OHDA mice relative to those from controls across a range of stimulation intensities (Fig. 1, C and D). However, the amplitude of thalamo-PT oEPSCs from the DA-intact contralateral hemisphere was unaltered (fig. S2), suggesting that the reported compensation in the contralateral hemisphere of hemiparkinsonism may not occur at the thalamocortical connections (*33*). Thus, studies in the rest of this work will focus on synaptic adaptations in the ipsilateral hemisphere. Moreover, the amplitude of oEPSCs in the IT neurons (thalamo-IT) was similar between 6-OHDA mice and controls (Fig. 1, E and F). These data suggest that chronic SNc DA degeneration selectively decreases synaptic strength of the motor thalamic excitation to M1 PT neurons in the hemisphere with lesion.

Multiple long-range cortical and thalamic excitatory inputs converge on M1 pyramidal neurons (*8*). Thus, we examined whether SNc DA degeneration also affects other excitatory inputs to PT neurons. We injected AAV-hSyn-ChR2(H134R)-eYFP into the sensory cortical (SC) regions (Fig. 1G) and retrobeads into the pons to retrogradely label PT neurons. The amplitude of oEPSCs from SC to PT neurons (SC-PT) was not altered between 6-OHDA mice and controls (Fig. 1, H and I). These data suggest that DA degeneration induces input-specific changes to M1 PT neurons. Together, chronic DA degeneration triggers cell subtype– and input-specific adaptions in M1 local circuits.

### SNc DA degeneration decreases the number of functional thalamic inputs to PT neurons

**Fig. 2.**
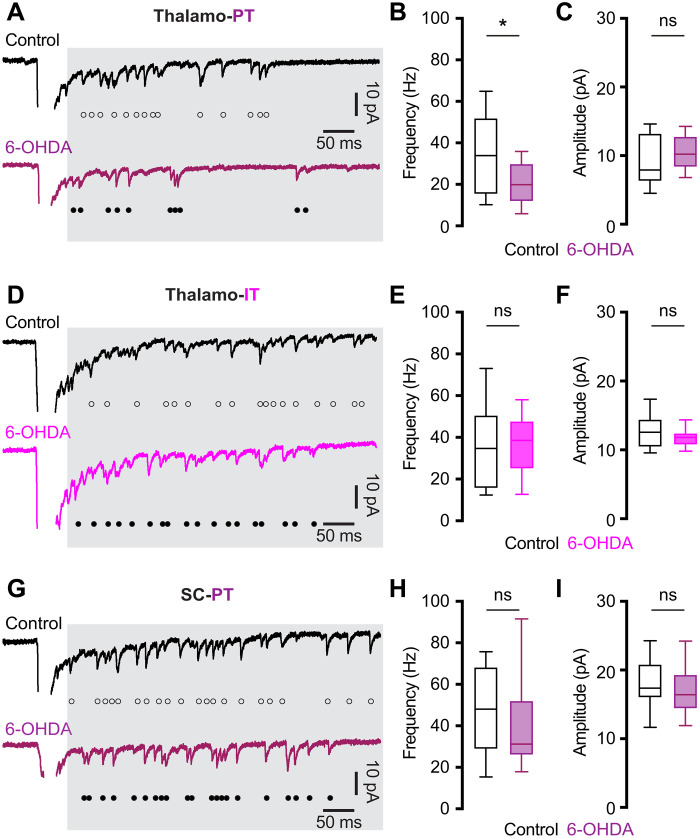
SNc DA degeneration decreases the number of functional thalamic inputs to PT neurons. (**A**) Representative traces showing optogenetically evoked Sr^2+^-EPSCs of PT neurons from controls and 6-OHDA mice. (**B** and **C**) Box plots showing a reduced frequency [B, controls = 34 [15.5 to 52] Hz, *n* = 20 neurons per three mice; 6-OHDA = 20 [12 to 30] Hz, *n* = 20 neurons per four mice; *P* = 0.02, Mann-Whitney *U* (MWU) test] and unaltered amplitude (C, controls = 7.95 [6.3 to 13.2] pA, *n* = 20 neurons per three mice; 6-OHDA = 10.2 [8.4 to 12.8] pA, *n* = 20 neurons per four mice; *P* = 0.33, MWU) of Sr^2+^-EPSCs at thalamo-PT synapses from 6-OHDA mice relative to controls. (**D**) Representative traces showing optogenetically evoked Sr^2+^-EPSCs at thalamo-IT synapses from controls and 6-OHDA mice. (**E** and **F**) Box plots showing unaltered frequency (E, controls = 34.8 [15.7 to 50.5] Hz, *n* = 22 neurons per three mice; 6-OHDA = 38.4 [24.9 to 47.6] Hz, *n* = 22 neurons per three mice; *P* = 0.7, MWU) and amplitude (F, control = 12.6 [10.5 to 14.4] pA; 6-OHDA = 11.8 [10.8 to 12.4] pA; *n* = 22 neurons per three mice; *P* = 0.34, MWU) of Sr^2+^-EPSCs at thalamo-IT synapses between groups. (**G**) Representative traces showing optogenetically evoked Sr^2+^-EPSCs at SC-PT synapses from controls and 6-OHDA mice. (**H** and **I**) Box plots showing unaltered frequency (H, controls = 48 [29 to 68] Hz, *n* = 20 neurons per three mice; 6-OHDA = 31.2 [26 to 52] Hz, *n* = 19 neurons per three mice; *P* = 0.6, MWU) and amplitude (I, control = 17 [16 to 20.8] pA, *n* = 20 neurons per three mice; 6-OHDA = 16.5 [14.4 to 19.3] pA, *n* = 19 neurons per three mice; *P* = 0.5, MWU) of Sr^2+^-EPSCs at SC-PT synapses between groups. Open and filled circles in (A), (D), and (G) indicate individual events of asynchronous EPSCs. All data are reported as median and interquartile range (in square brackets).

DA degeneration did not alter the paired pulse ratio at thalamo-PT, thalamo-IT, or SC-PT synapses (fig. S3), suggesting that the selective reduction of thalamic excitation to PT neurons was not due to a lower initial release probability at thalamo-PT synapses. To understand presynaptic mechanisms underlying synaptic adaptations of M1 circuits following the loss of SNc DA neurons, we assessed the quantal properties of thalamic glutamatergic neurotransmission to PT and IT neurons (*30*, *34*, *35*). Replacing extracellular Ca^2+^ with Sr^2+^ (2 mM), we found a substantial reduction of the frequency, but not the amplitude, of optogenetically evoked asynchronous EPSCs (Sr^2+^-EPSCs) at the thalamo-PT synapses from 6-OHDA mice relative to controls (Fig. 2, A to C). In contrast, neither the frequency nor the amplitude of Sr^2+^-EPSCs at the thalamo-IT synapses was altered in 6-OHDA mice relative to controls (Fig. 2, D to F). Moreover, neither the frequency nor the amplitude of Sr^2+^-EPSCs at SC-PT synapses was altered by DA depletion (Fig. 2, G to I). These data further support that SNc DA degeneration selectively decreases the connection strengthen of thalamic projection to PT neurons, at least partially due to fewer functional thalamo-PT connections.

### SNc DA degeneration induces structural changes at thalamo-PT synapses

**Fig. 3.**
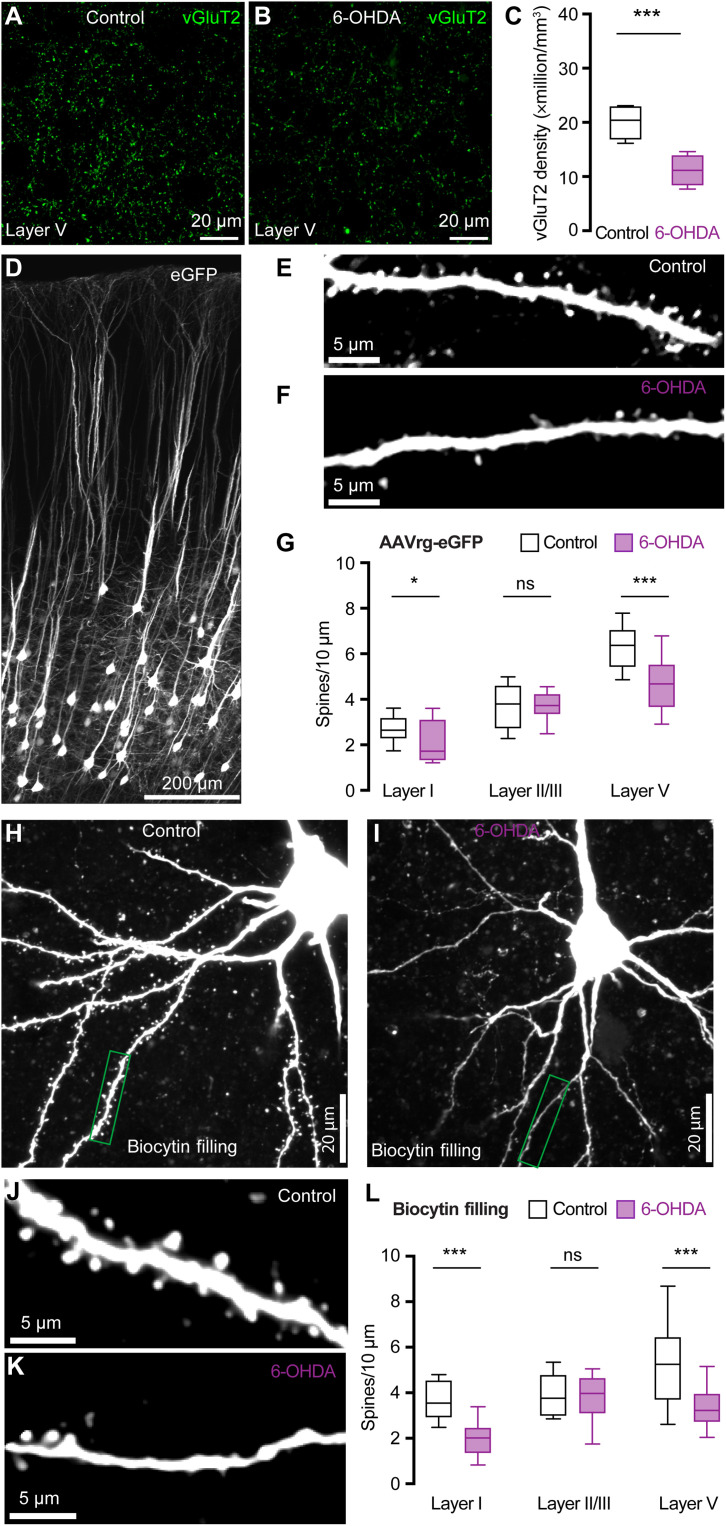
SNc DA degeneration induces structural changes at thalamo-PT synapses. (**A** and **B**) Representative confocal images of vGluT2 immunoreactivity in the layer V of M1 from controls and 6-OHDA mice. (**C**) Summarized results (control = 20.4 [16.9 to 22.9] million/mm^3^; 6-OHDA = 11.2 [8.5 to 13.9] million/mm^3^, *n* = 20 slices per three mice for each group; *P* < 0.0001, MWU). (**D** to **F**) Representative images of eGFP-labeled neurons in M1 (D) and segments of basal dendrites from controls (E) and 6-OHDA mice (F). (**G**) Summarized results (layer V: control = 6.4 [5.4 to 7.1]/10 μm, *n* = 53 segments per three mice; 6-OHDA = 4.7 [3.6 to 5.5]/10 μm, *n* = 47 segments per three mice; *P* < 0.001, MWU; layer I: controls = 2.6 [2.3 to 3.2]/10 μm, *n* = 24 segments per three mice; 6-OHDA = 1.7 [1.3 to 3.1]/10 μm, *n* = 25 segments per three mice; *P* = 0.02, MWU; layer II/III: controls = 3.6 [2.8 to 4.4]/10 μm, *n* = 38 segments per three mice; 6-OHDA = 3.5 [3.0 to 4.2]/10 μm, *n* = 36 segments per three mice; *P* = 0.7, MWU). (**H** and **I**) Representative images of biocytin-filled PT neurons from controls and 6-OHDA mice. (**J** and **K**) Representative images of segments of dendrites from controls and 6-OHDA mice [indicated by green boxes in (H) and (I)]. (**L**) Summarized results (layer V: control = 5.2 [3.7 to 6.4]/10 μm, *n* = 34 segments per three mice; 6-OHDA = 3.2 [2.7 to 4.0]/10 μm, *n* = 40 segments per three mice; *P* < 0.001, MWU; layer I: controls = 3.6 [2.9 to 4.6]/10 μm, *n* = 24 segments per three mice; 6-OHDA = 2.0 [1.3 to 2.5]/10 μm, *n* = 24 segments per three mice; *P* < 0.001, MWU; layer II/III: controls = 3.8 [3.0 to 4.8]/10 μm, *n* = 24 segments per three mice; 6-OHDA = 4.0 [3.1 to 4.7]/10 μm, *n* = 22 segments per three mice; *P* = 0.6, MWU).

To assess how loss of DA alters thalamocortical innervation of M1 PT neurons, brain sections from control and 6-OHDA mice were processed for immunohistochemical detection of vesicular glutamate transporter 2 (vGluT2), a marker of thalamic axon boutons in M1. Stereological methods were used to quantify vGluT2-immunoreactive (vGluT2-ir) puncta in the layer V of M1. Consistent with findings from 1-methyl-4-phenyl-1,2,3,6-tetrahydropyridine (MPTP)–treated nonhuman primates (*24*), the density of vGluT2-ir puncta in layer V was significantly decreased in slices from 6-OHDA mice relative to those from controls (Fig. 3, A to C). Thus, DA depletion likely induces a loss of presynaptic thalamic axon terminals in M1, although a potential decrease in vGluT2 expression cannot be excluded.

To interrogate postsynaptic structural changes associated with DA depletion, we injected either adeno-associated virus retrograde serotype (AAVrg)–enhanced green fluorescent protein (eGFP) into the pontine nuclei to label M1 PT neurons using eGFP (Fig. 3, D to G) or retrobeads into the pontine nuclei followed by ex vivo biocytin filling via patch pipettes for morphology studies (Fig. 3, H to L). The density of dendritic spines of PT neurons across M1 layers was assessed in controls and 6-OHDA mice. Experiments using two different experimental approaches showed consistent results. Specifically, the spine density of the basal dendrites in layer V and the apical dendrites in layer I from 6-OHDA mice decreased significantly relative to those from controls (Fig. 3, E to G and J to L). This is consistent with the preferential motor thalamic projections to layers I and V (*5*, *8*, *24*). In contrast, the spine density of apical dendrites of PT neurons in layer II/III was not altered following the loss of SNc DA neurons (Fig. 3, G and L). These data are consistent with the unaltered SC-PT excitation in 6-OHDA mice (Figs. 1 and 2) and the earlier reports showing preferential SC projections to layer II/III (*8*). For comparison, AAVrg-eGFP–labeled IT neurons showed no change in the spine density of either basal or apical dendrites across cortical layers (fig. S4). Thus, SNc DA degeneration induces loss of dendritic spines of PT neurons those are mainly targeted by the thalamic inputs, but not those targeted by sensory inputs. The presynaptic and postsynaptic morphological changes further support our hypothesis that selective impairments of thalamocortical synapses onto M1 PT neurons occur in parkinsonism.

### SNc DA degeneration decreases the effectiveness of thalamic driving of M1 PT neuronal firing

As reported in the somatosensory cortex (*36*), optogenetically synchronized mTh excitation could effectively drive M1 PT neuronal firing in control animals, which was mediated by both AMPARs and NMDARs (fig. S5, A and B). Following SNc DA neurodegeneration, the composition of glutamatergic receptors at the thalamo-PT synapses, measured as the NMDA/AMPA ratio, was not altered (fig. S5, C and D), indicating that synchronous thalamic excitation remains capable to drive PT neuronal firing.

Given the prevalence of abnormally synchronized thalamocortical activity in parkinsonism (*16*, *37*, *38*), it is therefore plausible to hypothesize that thalamic inputs drive the aberrant pattern of PT neuronal activity through the strengthened remaining thalamo-PT connections at dendrites and spines, the excitability of which cannot be accurately measured using whole-cell voltage-clamp recordings. To assess this possibility, we recorded retrogradely labeled PT and IT neurons under current-clamp mode and compare the effectiveness of thalamic and cortical inputs in driving neuronal firing in both control and 6-OHDA mice. A 20-Hz optogenetic stimulation (1-ms duration) was delivered to activate ChR2-expressed thalamic or SC axons in M1.

**Fig. 4.**
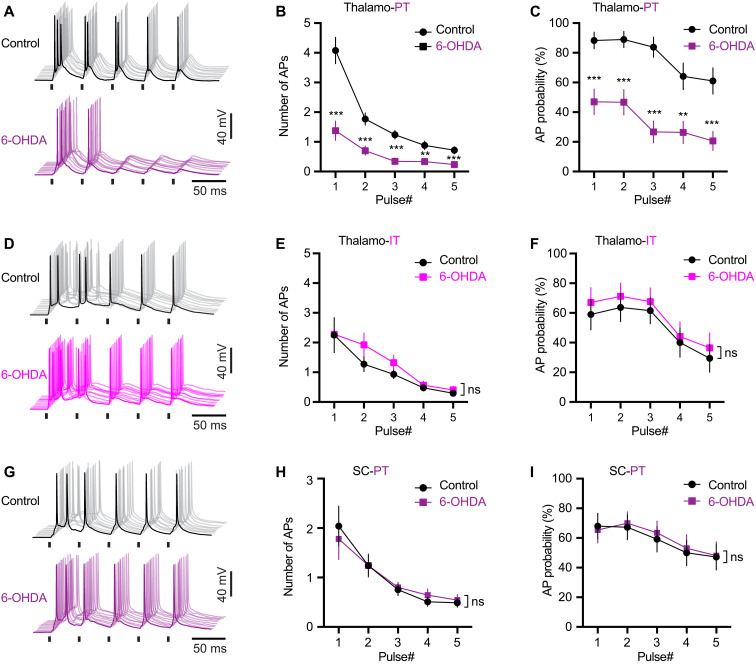
SNc DA degeneration decreases the effectiveness of thalamic driving of M1 PT neuronal firing. (**A**) Representative traces of AP firing of M1 PT neurons from controls and 6-OHDA mice in response to 20-Hz optogenetic stimulation of thalamocortical inputs. AP traces from 10 trials were aligned for both groups. (**B** and **C**) Summarized results show that both the number (B) and probability (C) of synaptically generated APs in M1 PT neurons decreased significantly in 6-OHDA mice relative to controls. Mixed effects model followed by Sidak’s tests, *P* < 0.0001 for group difference. Control = 29 neurons per three mice; 6-OHDA = 33 neurons per four mice. (**D**) Representative traces of AP firing of M1 IT neurons from controls and 6-OHDA mice in response to 20-Hz optogenetic stimulation of thalamocortical inputs. (**E** and **F**) Summarized results show that the number (E) and probability (F) of synaptically generated APs in M1 IT neurons was not altered in 6-OHDA mice relative to controls. Mixed effects model, *P* = 0.56 for group difference on AP probability; *P* = 0.36 for group difference on the number of APs; control = 19 neurons per three mice for each group. (**G**) Representative traces of AP firing of M1 PT neurons from controls and 6-OHDA mice in response to 20-Hz optogenetic stimulation of SC inputs. (**H** and **I**) Summarized results show that both the number (H) and probability (I) of synaptically generated APs in M1 PT neurons upon stimulation of cortical inputs were not altered in 6-OHDA mice relative to controls. Mixed effects model, *P* = 0.76 for group difference on AP probability; *P* = 0.97 for group difference on the number of APs; control = 25 neurons per three mice; 6-OHDA = 26 neurons per three mice. Light intensity was 2.3 mW/mm^2^ for all patterning experiments. Short vertical bars in (A), (D), and (G) indicate the period when optical stimulation was delivered.

The effectiveness of thalamic driving of PT neuronal firing decreased significantly in slices from 6-OHDA mice relative to those from controls, as reflected by fewer APs per stimulation and a decreased AP firing probability (Fig. 4, A to C). In contrast, we did not detect alterations in the effectiveness of thalamo-IT or SC-PT synaptic driving of neuronal firing between groups (Fig. 4, D to F and G to I). Consistent with the input- and cell subtype–specific changes in synaptic strength, these data further suggest that loss of DA selectively disrupts the functional connectivity between the motor thalamus and PT neurons. The results also suggest that monosynaptic thalamic inputs are not sufficient to synchronize PT neuronal firing in bursts under parkinsonian state.

### Thalamic and SC inputs to PT neurons exhibit distinct composition of glutamatergic receptors

**Fig. 5.**
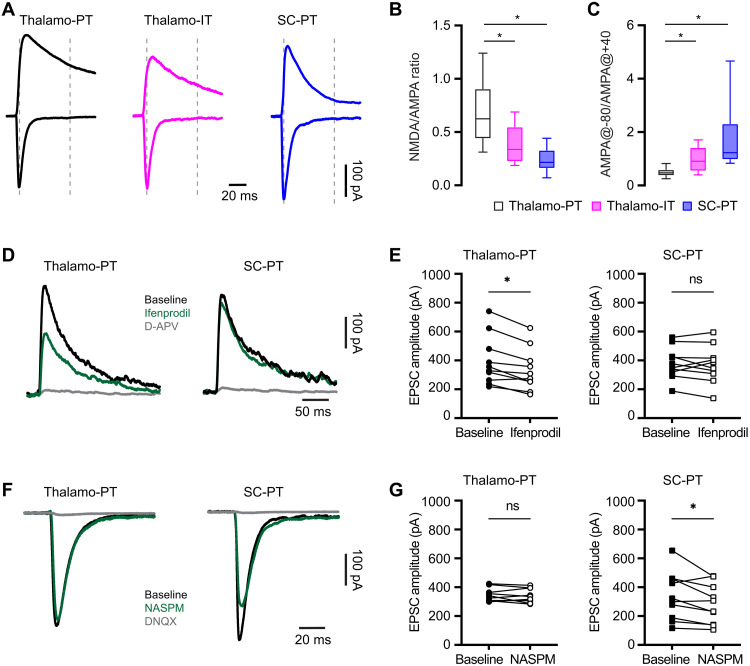
Thalamic and SC inputs to PT neurons exhibit distinct postsynaptic receptor compositions. (**A**) Representative traces of AMPAR- and NMDAR-mediated responses from different synapses. (**B** and **C**) Box plots showing differences in NMDA/AMPA ratios (thalamo-PT = 0.62 [0.44 to 0.91], *n* = 34 neurons per four mice; thalamo-IT = 0.34 [0.22 to 0.55], *n* = 19 neurons per three mice; SC-PT = 0.22 [0.16 to 0.33], *n* = 25 neurons per three mice; *P* < 0.0001; Kruskal-Wallis test followed by Dunn’s test) and AMPAR rectification properties (thalamo-PT = 0.47 [0.37 to 0.60], *n* = 34 neurons per four mice; thalamo-IT = 0.91 [0.54 to 1.4], *n* = 25 neurons per three mice; SC-PT = 1.23 [0.97 to 2.32], *n* = 19 neurons per three mice; *P* < 0.0001; Kruskal-Wallis test followed by Dunn’s test) of different synapses in M1. (**D** and **E**) Different sensitivities of NMDARs-EPSCs at thalamic and SC synapses to ifenprodil. (D) Representative traces of isolated NMDARs-mediated EPSCs. (E) Connected symbols showing the effects of ifenprodil to the amplitude of NMDARs-EPSCs at thalamo-PT {left, baseline = 342 [256 to 516] pA, ifenprodil = 292 [234 to 428] pA; *n* = 10 neurons per four mice; *P* = 0.004, Wilcox signed rank (WSR)} and SC-PT (right, baseline = 363 [313 to 452] pA, ifenprodil = 366 [296 to 443] pA; *n* = 10 neurons per three mice; *P* = 0.6, WSR) synapses. (**F**) Representative traces of AMPARs-EPSCs from thalamo-PT and SC-PT synapses at baseline, in NASPM, and in NASPM plus DNQX. (**G**) Εffects of NASPM to the amplitude of AMPARs-EPSCs at thalamo-PT (left, baseline = 333 [305 to 378] pA; NASPM = 327 [301 to 395] pA; *n* = 10 neurons per four mice; *P* = 0.6, WSR) and SC-PT synapses (right, baseline = 311 [181 to 459] pA; NASPM = 269 [106 to 419] pA; *n* = 10 neurons per four mice; *P* = 0.02, WSR).

NMDARs are critical for thalamocortical synaptic integration and plasticity and contribute to pathological processes in neurological disorders. Thus, we assessed whether NMDAR activation mediates the selective impairment of thalamo-PT inputs, but not the thalamo-IT or SC-PT synapses, following the loss of DA. Specifically, we found that the NMDA/AMPA ratio at the thalamo-PT synapses, relative to thalamo-IT synapses and SC-PT synapses, was significantly higher (Fig. 5, A and B). These data indicate that NMDARs are functionally enriched at thalamo-PT synapses relative to SC-PT or thalamo-IT synapses. In addition, there was a stronger inward rectification of AMPARs at SC-PT synapses relative to thalamic projections to PT or IT neurons (Fig. 5, A and C). These data indicate that GluA2-lacking, Ca^2+^-permeable AMPARs are enriched at SC-PT synapses relative to those from the motor thalamus to M1 PT or IT neurons in control mice.

Next, we used a pharmacological approach to further confirm the input-specific composition of glutamatergic receptors in PT neurons. A large body of evidence suggests that GluN2B subunit–containing NMDA receptors with larger Ca^2+^ conductance enriched at thalamocortical synapses in the prefrontal cortex and play a critical role in pathological processes in neurological diseases (*39*, *40*). Consistently, a noncompetitive antagonist of GluN2B-containing NMDARs ifenprodil (3 μM) significantly and consistently reduced the amplitude of NMDARs-EPSCs at thalamo-PT synapses but did not produce consistent effect to the NMDAR-EPSC amplitude of SC-PT synapses (Fig. 5, D and E). In addition, to quantitatively assess the functional contribution of GluA2-lacking, Ca^2+^-permeable AMPARs at different inputs, a selective antagonist 1-naphthyl acetyl spermine trihydrochloride (NASPM) was used. Bath application of NASPM (100 μμ) consistently decreased the amplitude of AMPARs-EPSCs of SC-PT synapses but did not produce significant effect to AMPARs-EPSCs at thalamo-PT synapses (Fig. 5, F and G). Together, we conclude that GluN2B-containing NMDARs are enriched at thalamo-PT synapses and that GluA2-lacking, Ca^2+^-permeable AMPARs functionally contribute more to SC-PT transmission in physiological state.

### NMDARs mediate the decreased thalamocortical transmission to M1 PT neurons

**Fig. 6.**
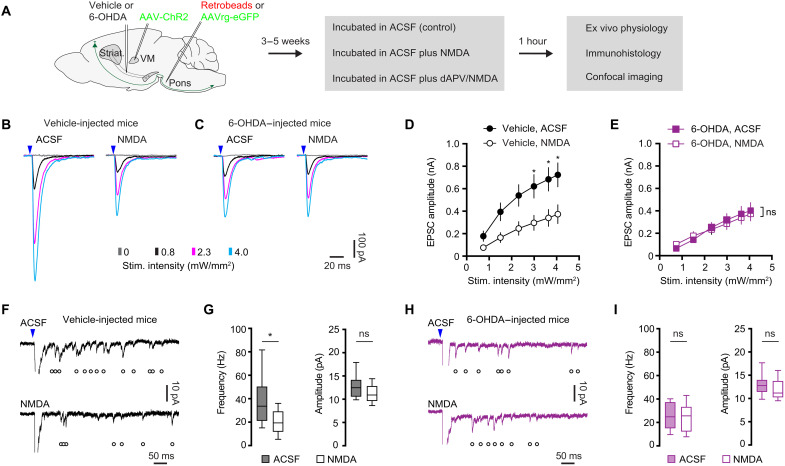
NMDAR activation decreases thalamo-PT transmission. (**A**) Experimental design. (**B** and **C**) Representative traces of optogenetically evoked thalamo-PT EPSCs from vehicle-injected controls (B) and 6-OHDA–injected mice (C), which were either incubated in normal ACSF (control) or ACSF-containing NMDA. (**D** and **E**) Summarized results show that NMDA incubation decreased the amplitude of oEPSCs from vehicle-injected controls (D, group effect, *P* < 0.0001, mixed effects model followed by Sidak’s tests), but not those from 6-OHDA–injected mice (E, group effect, *P* > 0.79, mixed effects model). (**F** and **G**) NMDA incubation decreased the frequency but not the amplitude of optogenetically evoked Sr^2+^-EPSCs from vehicle-injected controls. (F) Representative traces of Sr^2+^-EPSCs. (G) Summarized results (frequency of Sr^2+^-EPSCs in vehicle-injected mice, control = 33.6 [21.1 to 50.4] Hz, *n* = 24 neurons per three mice; NMDA = 19.4 [11.8 to 29.2] Hz; *n* = 24 neurons per three mice; *P* = 0.001, MWU; amplitude of Sr^2+^-EPSCs in vehicle-injected mice, control = 12.5 [10.5 to 14.2] pA, NMDA = 10.9 [9.6 to 12.9] pA; *n* = 24 neurons per three mice, *n* = 24 neurons per three mice; *P* = 0.1, MWU). (**H** and **I**) NMDA incubation did not alter the frequency or the amplitude of optogenetically evoked Sr^2+^-EPSCs from 6-OHDA–injected mice. (H) Representative traces of Sr^2+^-EPSCs. (I) Summarized results (frequency of Sr^2+^-EPSCs in 6-OHDA mice, control = 24.8 [15 to 37.4] Hz, *n* = 17 neurons per three mice; NMDA = 25.6 [12.2 to 33.4] pA, *n* = 17 neurons per three mice; *P* = 0.6, MWU; amplitude of Sr^2+^-EPSCs in 6-OHDA mice, control = 12.9 [11.3 to 14.0] pA, *n* = 17 neurons per three mice; NMDA = 11.2 [10.3 to 13.8] Hz, *n* = 27 neurons per three mice; *P* = 0.21, MWU).

Next, given the enrichment of NMDARs at thalamo-PT synapses, we further assessed whether NMDAR activation mediates the decreased thalamo-PT synaptic strength in parkinsonism. Studies, including our own recent work, report that activation of NMDARs by exogenously application of NMDA is sufficient to mimic NMDAR-dependent pathological processes in BG nuclei (*34*, *41*, *42*). When we incubated brain slices with exogenous NMDA (25 μM) for 1 hour, the treatment did not decrease cell density of M1 or affect the cellular excitability of PT neurons (fig. S6), indicating that the NMDA treatment was not excitotoxic (*34*). Brain slices from controls and 6-OHDA mice were then incubated with (i) normal artificial cerebrospinal fluid (ACSF), (ii) NMDA-containing ACSF (25 μM), or (iii) D-2-amino-5-phosphonovaleric acid (D-APV)/NMDA-containing ACSF for 1 hour (*34*), before physiological and anatomical studies (Fig. 6A). In brain slices from vehicle-injected control mice, ex vivo NMDA incubation reduced the amplitude of thalamo-PT oEPSCs but did not alter NMDA/AMPA ratio and AMPA rectification index at thalamo-PT synapses (Fig. 6, B and D). The suppression of thalamo-PT oEPSCs by NMDA treatment could be abolished by D-APV coincubation (fig. S7). In addition, ex vivo NMDA incubation did not alter the amplitude of thalamo-IT oEPSCs (fig. S8), further arguing for a synapse- and cell subtype–specific effect of the NMDA treatment. Together, these data indicate that NMDAR stimulation induces synapse- and cell subtype–specific changes in M1 circuits and that such specificity might be due to compartment-specific expression of NMDAR subunits (Fig. 5).

If NMDARs and the associated signaling cascade become activated upon loss of SNc DA neurons, contributing to the reduced thalamo-PT excitation, then a corollary would be that the effects of ex vivo NMDAR stimulation to thalamo-PT oEPSCs (Fig. 6, B and D) should be occluded in slices from 6-OHDA mice. Consistently, ex vivo NMDA incubation had no effect to the amplitude of thalamo-PT oEPSCs from 6-OHDA mice (Fig. 6, C and E). These data suggest that excessive NMDAR activation is sufficient to decrease the thalamic excitation to PT neurons from control mice and that the associated signaling cascade likely mediates the reduced thalamic excitation to PT neurons in vivo following DA depletion.

Loss of DA decreases the number of functional thalamocortical connections to PT neurons (Figs. 2 and 3). Thus, we further studied the impact of exogenous NMDA incubation on Sr^2+^-induced quantal glutamate release at thalamocortical synapses to PT neurons. In slices from vehicle-injected controls, NMDA incubation significantly decreased the frequency, but not the amplitude of optogenetically evoked thalamo-PT Sr^2+^-EPSCs (Fig. 6, F and G). In contrast, in slices from 6-OHDA mice, NMDA incubation did not alter the frequency or the amplitude of optogenetically evoked thalamo-PT Sr^2+^-EPSCs (Fig. 6, H and I). The above data suggest that NMDAR activation reduces the number of functional thalamocortical inputs to M1 PT neurons, but this effect was occluded in 6-OHDA mice.

**Fig. 7.**
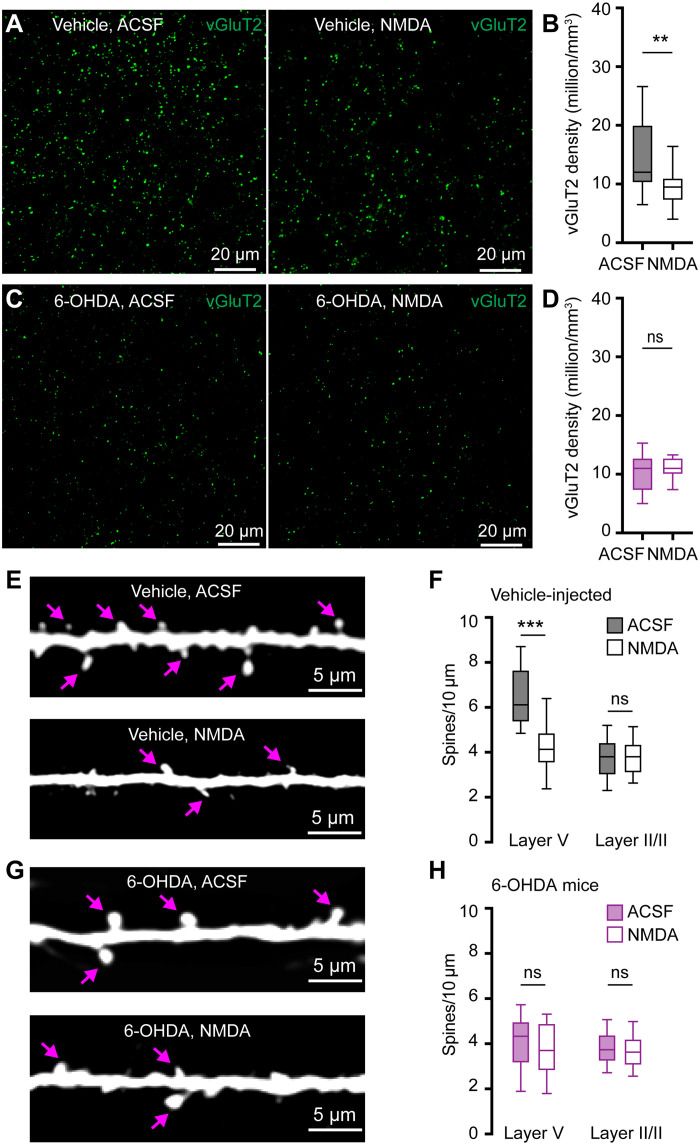
NMDAR activation induces structural changes of thalamo-PT synapses. (**A** and **B**) NMDA incubation decreases vGluT2 density in slices from vehicle injected controls. (A) Representative images. (B) Summarized results of controls (ACSF = 12 [10 to 14.8] million/mm^3^, *n* = 20 samples per three mice; NMDA = 9 [7 to 10.8] million/mm^3^, *n* = 16 samples per three mice; *P* = 0.0016, MWU). (**C** and **D**) NMDA incubation does not alter vGluT2 density in slices from 6-OHDA mice. (C) Representative images. (D) Summarized results of 6-OHDA mice (ACSF = 11 [8 to 12] million/mm^3^, NMDA = 11 [9 to 12] million/mm^3^, 20 samples per three mice for each group; *P* = 0.7, MWU). (**E**) Representative images of layer V dendritic spines from ACSF- and NMDA-treated slices of controls. (**F**) Summarized results show that NMDA incubation reduced layer V spines in slices of controls (ACSF = 6 [5.4 to 7.6] spines/10 μm, *n* = 24 samples per three mice; and NMDA = 4.1 [3.5 to 4.9] spines/10 μm, *n* = 25 samples per three mice; *P* < 0.0001, MWU) but did not alter the density of layer II/III apical dendritic spine (ACSF = 3.8 [3 to 4.4] spines/10 μm; NMDA = 3.8 [3.1 to 4.3] spines/10 μm; 36 samples per three mice for each group; *P* = 0.7, MWU). (**G**) Representative images of layer V dendritic spines from ACSF- and NMDA-treated slices of 6-OHDA mice. (**H**) Summarized results show that NMDA incubation did not alter layer V or II/III spine densities in slices from 6-OHDA mice (layer V: ACSF = 4.3 [3.2 to 5] spines/10 μm, *n* = 29 samples per three mice; NMDA = 3.7 [2.8 to 4.9] spines/10 μm, *n* = 31 samples per three mice; *P* = 0.4, MWU; layer II/III: ACSF = 3.7 [3.2 to 4.4] spine/10 μm; NMDA = 3.6 [3.1 to 4.2] spines/10 μm, 36 samples per three mice for each group; *P* = 0.45, MWU). Arrows in (E) and (G) indicate representative spines.

Next, we studied whether ex vivo NMDA incubation would induce structural changes at thalamo-PT synapses. A separate set of brain slices from controls and 6-OHDA mice were incubated with ACSF or NMDA-containing ACSF for 1 hour and then fixed with 4% paraformaldehyde (PFA) for immunohistochemistry studies. We found that ex vivo NMDA incubation robustly decreased vGluT2 densities in layer V of M1 of controls, relative to those incubated in ACSF (Fig. 7, A and B). However, NMDA incubation did not alter vGluT2 densities in layer V from 6-OHDA mice relative to those treated with normal ACSF (Fig. 7, C and D). Postsynaptically, NMDA incubation decreased the spine densities of basal dendrites in layer V of retrogradely labeled PT neurons in controls but did not alter the spine density of apical dendrites in layer II/III (Fig. 7, E and F). In 6-OHDA mice, ex vivo NMDA incubation did not alter spine densities of PT neurons in either layer V or layer II/III (Fig. 7, G and H). Together, our physiological and anatomical results suggest that excessive NMDAR activation is sufficient to decrease thalamocortical connection to M1 PT neurons and that the NMDARs-mediated mechanisms are synapse specific, likely underlying the decreased thalamic excitation to PT neurons in 6-OHDA mice.

### Disrupting BG outputs rescues the decreased thalamo-PT transmission in parkinsonism

Next, we attempted to understand the interaction between M1 adaptations and the abnormal BG output in parkinsonism. Given that disrupting BG output by DA medications or functional surgery effectively improves motor function in both PD subjects and parkinsonian animals (*43*–*45*), it has been proposed that prevention of abnormal BG activity from propagating to the downstream motor regions (e.g., the thalamocortical network) would allow them to perform normal function in motor control and there be beneficial (*46*, *47*). Therefore, we suppressed the SNr output using inhibitory designer receptors exclusively activated by designer drugs (DREADDs) to assess how such manipulation affects thalamocortical adaptations in both controls and DA-depleted mice.

**Fig. 8.**
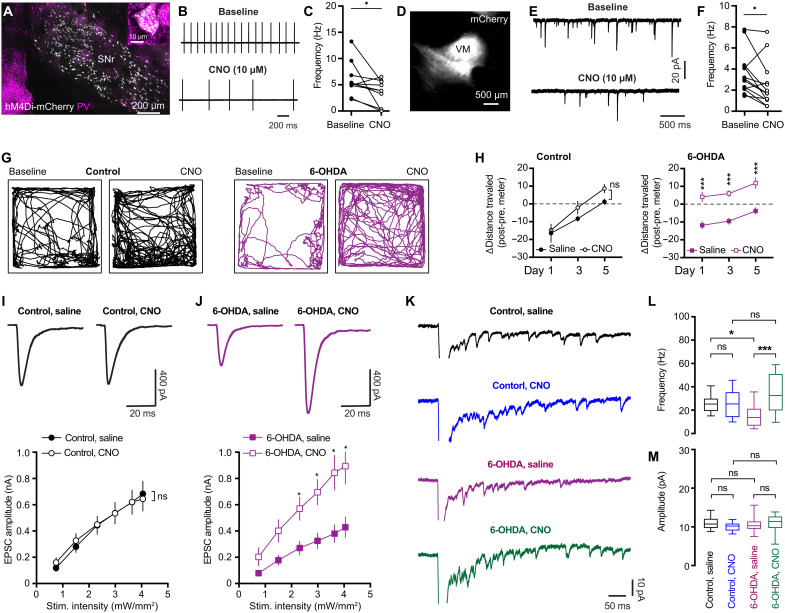
Disrupting BG output rescues thalamo-PT adaptations in parkinsonism. (**A**) hM4Di-mCherry expression in PV^+^ SNr neurons. (**B** and **C**) hM4Di activation decreased SNr neuronal firing. (B) Examples. (C) Summarized results (baseline = 5.2 [4.3 to 7.5] Hz, CNO = 3.9 [0.27 to 5.6] Hz; *n* = 10 neurons per three mice; *P* = 0.02, WSR). (**D**) Example of mCherry-labeled SNr axon terminals in mTh. (**E** and **F**) hM4Di activation reduced sIPSCs frequency of thalamic neurons (baseline = 3.1 [2.1 to 4.3] Hz, CNO = 1.7 [1.1 to 3.2] Hz; *n* = 13 neurons per three mice; *p* = 0.02, WSR). (**G** and **H**) hM4Di activation increased locomotion of 6-OHDA mice. (G) Representative track plots on day 5. (H) Summarized results showing the difference of distance traveled between pre- and postsaline/CNO injections. (**I** and **J**) hM4Di activation did not alter the amplitude of thalamo-PT EPSCs in controls (I, *P* = 0.9) but increased the amplitude of thalamo-PT EPSCs in 6-OHDA mice (J, *P* = 0.007, mixed effects model). Top: Examples. Bottom: Summarized results. Eighteen to 24 neurons per three to four mice for each group. (**K**) Representative traces of Sr^2+^-EPSCs in controls and 6-OHDA mice treated with saline or CNO. (**L**) Summarized results showing CNO injection did not alter the frequency of Sr^2+^-EPSCs from controls (control/saline = 25 [19 to 30] Hz, control/CNO = 25 [14 to 35] Hz; *P* > 0.9) but increased the frequency of Sr^2+^-EPSCs from 6-OHDA mice (6-OHDA/saline = 13.8 [6.7 to 21.3] Hz, 6-OHDA/CNO = 32.4 [19.6 to 50.8] Hz, *P* = 0.0001). (**M**) CNO injection did not alter the amplitude of Sr^2+^-EPSCs in either controls (control/saline = 10.7 [9.7 to 12.1] pA, *n* = 18 neurons per three mice; control/CNO = 10.2 [9.1 to 10.9] pA, *n* = 18 neurons per three mice; *P* = 0.8) or 6-OHDA mice (6-OHDA/saline = 10.3 [9.5 to 11.4] pA, *n*= 22 neurons per three mice; 6-OHDA/CNO = 11.4 [9.7 to 12.7] pA, *n* = 19 neurons per three mice; *P* = 0.95). Kruskal-Wallis and Dunn’s tests.

Because parvalbumin-expressing (PV^+^) GABAergic cells account for majority of SNr projection neurons (*7*), we unilaterally injected PV-Cre knock-in mice with vehicle or 6-OHDA into the MFB plus AAV9-hSyn-FLEX-hM4Di-mCherry into the SNr of the same hemisphere to chemogenetically manipulate BG outputs (Fig. 8A). Immunofluorescence staining showed the selective expression of hM4Di (Gi)-DREADDS-mCherry in SNr PV^+^ cells (Fig. 8A) and a preferred projection of mCherry-labeled axon terminals in the VM motor thalamus (Fig. 8D). There was no difference in the number of hM4Di-expressing cells in the SNr between 6-OHDA mice and controls. Bath application of DREADD agonist clozapine *N*-oxide (CNO; 10 μμ) effectively suppressed the frequency of autonomous firing of SNr neurons from 6-OHDA mice (Fig. 8, B and C) as well as the frequency of spontaneous inhibitory postsynaptic currents (sIPSCs) of VM thalamic neurons (Fig. 8, E and F).

PV-Cre mice with hM4Di expression in PV^+^ SNr neurons were subcutaneously injected with either CNO (1.0 mg/kg, at 12-hour interval between injections) or saline for five consecutive days to continuously disrupt the abnormal BG outputs or control for subcutaneous injections, respectively. Open field locomotor activities were measured on days 1, 3, and 5. Difference in the total distance traveled before and after injections was comparable between CNO-injected and saline-injected control mice (Fig. 8, G and H). This is perhaps because that CNO’s effects to unilateral SNr neuronal activity of DA intact mice were not sufficient to robustly affect general motor activity. As predicted, CNO injection substantially increased the locomotor activity of 6-OHDA mice relative to saline-injected mice (Fig. 8, G and H). These data indicate that the SNr output was effectively suppressed by hM4Di activation in 6-OHDA mice, leading to behavioral improvements (*48*, *49*). The improved locomotor activity following the SNr suppression has been reported to be mediated by disinhibiting pedunculopontine nucleus (PPN) (*50*). Given the extensive SNr collateral projections to the PPN and the motor thalamus (*7*), it is reasonable to posit that hM4Di activation could also disrupt GABAergic transmission from the SNr to the motor thalamus, which is supported by CNO-induced reduction of sIPSCs frequency recorded from the VM motor thalamic neurons (Fig. 8, D to F).

To determine whether disruption of pathological BG inputs to the mTh could rescue adaptive changes of M1 circuits, the same cohort of mice described above also received retrobeads injection into the pons to retrogradely label M1 PT neurons and AAV-ChR2(H134R)-eYFP injection into the mTh for optogenetic stimulation of thalamo-PT synapses. In vehicle-injected control mice, we found that chemogenetic suppression of SNr activity through repetitive CNO injection did not alter the amplitude of optogenetically evoked thalamo-PT oEPSCs (Fig. 8I). In contrast, repetitive CNO injection substantially increased the amplitude of optogenetically evoked thalamo-PT oEPSCs in 6-OHDA mice (Fig. 8J).

Further, we assessed the effects of chemogenetic suppression of SNr activity to quantal properties of thalamocortical transmission to PT neurons. Chemogenetic suppression of SNr activity through repetitive CNO injection did not alter either the frequency or the amplitude of optogenetically evoked Sr^2+^-EPSCs at the thalamo-PT synapses of vehicle-injected control mice (Fig. 8, K to M). In contrast, repetitive CNO injection increased the frequency, but not the amplitude, of optogenetically evoked Sr^2+^-EPSCs at the thalamo-PT synapses of 6-OHDA mice (Fig. 8, K to M). Together, these data indicate that chemogenetic suppression of BG outputs can rescue the decreased thalamo-PT synaptic transmission in 6-OHDA mice.

## DISCUSSION

The present study advances our understanding of motor cortical circuitry adaptations following the degeneration of SNc DA neurons. First, we demonstrated that DA depletion induces cell subtype– and input-specific down-regulation of thalamic excitation to M1 PT neurons (Fig. 1). Physiological and anatomical analyses showed that the weakened thalamocortical synaptic strength is likely associated with fewer thalamocortical connections (Figs. 2 to 4). Moreover, NMDARs are enriched at thalamocortical synapses to PT neurons (Fig. 5). Prolonged NMDAR activation ex vivo was sufficient to decrease thalamocortical excitation to PT neurons from control animals, but such effects were occluded in 6-OHDA mice (Figs. 6 and 7). These data suggest that excessive NMDAR activation in vivo is perhaps involved in mediating the decreased thalamocortical transmission after DA depletion. In addition, chemogenetic suppression of SNr activity can reverse the disrupted thalamo-PT synaptic transmission in parkinsonian mice (Fig. 8), suggesting that disrupting pathological BG output has the potential to rescue cortical circuitry function in parkinsonism.

Considering the complexity of cortical circuit and its plasticity mechanisms, the impact of BG dysfunction on M1 outputs should be more complicated than it has been proposed. Consistently, relative to IT neurons, PT neurons are functionally more vulnerable to DA degeneration, and their activities are robustly entrained by the pathological oscillations through the transition of the motor thalamus (*12*, *16*, *21*, *25*, *26*, *38*). We also have demonstrated a series of intrinsic (*26*) and thalamocortical adaptations in M1 of parkinsonian mice, mainly affecting PT neurons. Thus, both intrinsic and synaptic adaptations can contribute to an impaired thalamic driving of cortical PT neuronal firing, leading to reduced motor cortical outputs and impaired motor control in parkinsonian state (*12*, *14*, *17*, *21*, *30*, *51*, *52*).

Although the PT and IT neurons in M1 differ in their gene expression, morphology, and electrophysiological properties (*53*–*55*), the AMPAR-mediated excitatory inputs from the motor thalamus to them are comparable in healthy animals (*8*, *32*). Thus, we posited that additional cellular and circuit mechanisms are involved and contribute to cell subtype–selective changes in parkinsonian state. Accordingly, we found that a greater contribution of GluN2B-NMDARs to thalamo-PT transmission could be a critical mechanism, which may explain (i) the selective weakening of thalamo-PT synaptic strength (Fig. 1) and (ii) the selective loss of spines in layer V, but not in layer II/III, in slices from 6-OHDA mice (Fig. 3) or control slices with NMDAR stimulation (Fig. 7), as well as (iii) the unaltered thalamo-IT connections in slices prepared from 6-OHDA mice (Fig. 1) or slices from controls with NMDAR stimulation (fig. S8).

Our work demonstrates distinct levels of GluN2B-NMDARs at thalamic versus SC inputs to M1 PT neurons. This compartment- and input-specific expression of NMDARs at glutamatergic inputs to cortical neurons (i.e., pyramidal neurons or interneurons) has been reported (*39*, *56*, *57*). Earlier in vivo studies also indicated input-specific recruitments of AMPARs and NMDARs in mediating excitatory transmission of motor cortical PT neurons (*58*). The selective alterations of glutamatergic inputs to M1 in parkinsonism are consistent with findings from MPTP-treated parkinsonian monkeys, showing that abnormal loss of glutamatergic axon terminals in M1 selectively occurs in thalamocortical system, but not within the cortico-cortical projections (*24*).

Overstimulation of GluN2B-NMDARs by the excessive glutamate could be a key molecular mechanism underlying the weakened thalamo-PT synaptic connection in parkinsonism. M1 receives convergent glutamatergic projections from several cortical areas and subcortical regions (*8*). In parkinsonism, pathological patterns of pyramidal neuronal activity (e.g., enhanced burst firing) and synchronous glutamatergic inputs (e.g., thalamic afferents) are expected to significantly elevate local glutamate concentration (*16*, *36*, *38*), which in turn leads to GluN2B-NMDARs overstimulation and the associated signaling cascades (*34*, *39*, *59*, *60*), such as the ubiquitin-proteasome pathway (*59*, *61*) or cathepsin B-like protease activity that regulates actin-based cytoskeleton to mediate morphological changes (*62*). It is worth noting that adaptive changes at the distal dendrites of the layer I were not properly assessed in current physiology studies due to the limitations of experimental approaches, and therefore, future work is needed to explore this area using complementary approaches (e.g., voltage imaging technologies).

Impaired DA neuromodulation in M1 may also play a role in cortical dysfunction in PD (*63*). However, the mesocortical DA projections mainly arise from the ventral tegmental area in rodents (*64*), which are less affected in the model used in the present study. Also, the reduced thalamo-PT transmission could be rescued by chemogenetic suppression of BG output in DA-depleted mice. Thus, it is unlikely that the partial loss of DA neuromodulation in M1 plays a dominant role in the observations described in the present study. In contrast, the abnormal BG outputs might be the major pathological signals underlying the impaired cortical function and parkinsonian motor impairments. Of interests, chemogenetic suppression of BG outputs in DA-depleted animals could restore some aspects of thalamocortical function, and this finding could shed light on the circuit mechanisms underlying the symptomatic alleviation mediated by DA medications and functional surgery that occurs in PD (*46*, *47*).

## MATERIALS AND METHODS

### Experimental design

The prespecified objectives of this study were to determine synaptic changes at thalamic inputs to different subtypes of pyramidal neurons in the M1 in parkinsonism and define the underlying molecular and circuit mechanisms. To address these objectives, the present study combined ex vivo electrophysiology with AAV-mediated optogenetics and retrograde labeling to conduct cell type– and input-specific investigations of thalamic synaptic changes following chronic DA degeneration (a key parkinsonian pathology). Chemogenetics and behavior assays were used to manipulate circuit function and assess its associated behavioral effects. Stereotaxic surgeries, behavioral tests, and immunohistochemistry were conducted by two research technicians, and ex vivo physiology was conducted by a researcher who was blinded to treatment groups.

### Animals

Wild-type C57BL/6J mice of both sexes (3 to 4 months old, RRID:IMSR_JAX:000664) were obtained from the Van Andel Institute vivarium internal colony and used in the study. Homozygous PV-Cre knock-in mice were originally purchased from The Jackson Laboratory (stock no. 017320RRID: IMSR_JAX:017320, Bar Harbor, ME) and maintained at a C57/BL6J background in Van Andel Institute vivarium. Mouse genotyping was conducted through the service of Transnetyx Inc. (Cordova, TN). Mice were housed up to four animals per cage under a 12-hour/12-hour light/dark cycle with access to food and water ad libitum in accordance with NIH guidelines for care and use of animals. All animal studies were reviewed and approved by the Institutional Animal Care and Use Committee at Van Andel Institute (reference no. 22-02-006).

### Stereotaxic surgery for 6-OHDA and virus injections

Mice were randomly assigned to different treatment groups. Mice were mounted and secured in a stereotaxic frame (Kopf) under 2% isoflurane anesthesia. Throughout the procedure, body temperature of the mouse was maintained by a thermostatic heating pad. Once the skull was opened, small holes were drilled above the predetermined targets to inject: (i) 6-OHDA (3 to 4 μg) into the MFB [from bregma, anterior-posterior (AP) = −0.7 mm, mediolateral (ML) = +1.2 mm, and dorsoventral (DV) = −4.7 mm from the brain surface] to induce unilateral degeneration of nigrostriatal pathway or vehicle into the MFB to serve as vehicle-injected controls; (ii) retrobeads (200 nl, 10× dilution) into the ipsilateral pontine nuclei (from bregma, AP = −5.0 mm, ML = +0.6 mm, DV = −5.0 mm) or contralateral striatum (from bregma, AP = +0.2 mm, ML = −2.3 mm, DV = −2.9 mm) to retrogradely label PT and IT neurons, respectively; and (iii) AAV9-hSyn-ChR2(H134R)-eYFP (RRID:Addgene_127090, 300 nl at a titer of 3.6 × 10^12^ GC/ml) into the ventral subregion of the motor thalamus (from bregma, AP = −1.6 mm, ML = +0.8 mm, DV = −4.3 mm) to label thalamocortical axon terminals for optogenetics studies. Post hoc histology analysis showed that the AAV infected most of the ventral medial and ventral anteriolateral thalamus (VM/VAL; Fig. 1). A small cohort of mice also received pAAV9-hSyn-DIO-hM4D(Gi)-mCherry (RRID:Addgene_44362, 300 nl at a titer of 2.2 × 10^12^ GC/ml) injection into the SNr (from bregma, AP = −3.4 mm, ML = +1.4 mm, DV = −4.6 mm) for chemogenetic studies. For morphology studies, AAVrg-hSyn-eGFP (RRID:Addgene_50465, 300 nl at 2 × 10^13^ GC/ml) were injected into the pontine nuclei to retrogradely label PT neurons in the motor cortex. All injections were performed using a 10-μl syringe (Hamilton, Reno, NV) mounted on a motorized microinjector (Stoelting, Wood Dale, IL) at a speed of 100 nl/min. Details of the stereotaxic injection procedure can be found on Protocol.io (dx.doi.org/10.17504/protocols.io.rm7vzye28lx1/v1).

### Slice preparation for electrophysiology

Three to 4 weeks postinjections, brain slices were prepared for electrophysiology. Mice were anesthetized with avertin (250 to 300 mg/kg), followed by transcardial perfusion with ice-cold sucrose-based ACSF equilibrated with 95% O_2_/5% CO_2_ and containing 230 mM sucrose, 26 mM NaHCO_3_, 10 mM glucose, 10 mM MgSO_4_, 2.5 mM KCl, 1.25 mM NaH_2_PO_4_, 0.5 mM CaCl_2_, 1 mM sodium pyruvate, and 0.005 mM l-glutathione. Coronal brain sections (250 μm) containing the primary motor cortex were prepared using a vibratome (VT1200s, Leica, RRID:SCR_018453) in the same sucrose-based solution that was maintained at ~4°C using a recirculating chiller (FL300, Julabo, Allentown, PA). Slices were then held in ACSF equilibrated with 95% O_2_/5% CO_2_ and containing 126 mM NaCl, 26 mM NaHCO_3_, 10 mM glucose, 2 mM MgSO_4_, 2.5 mM KCl, 1.25 mM NaH_2_PO_4_, 1 mM sodium pyruvate, and 0.005 mM l-glutathione at 35°C for 30 min for recovery and then at room temperature until electrophysiology recordings or NMDA incubation. Details of slice preparation can be found on Protocols.io (dx.doi.org/10.17504/protocols.io.36wgqj2eovk5/v1).

### Brain slice physiology and optogenetics

Brain slices were placed in recording chamber with continuous perfusion of recording solution containing 126 mM NaCl, 26 mM NaHCO_3_, 10 mM glucose, 3 mM KCl, 1.6 mM CaCl_2_, 1.5 mM MgSO_4_, and 1.25 mM NaH_2_PO_4_. The solution was equilibrated with 95% O_2_/5%CO_2_ and maintained at 33° to 34°C using feedback controlled in-line heater (TC-324C, Warner Instruments). To assess synaptic strength, TTX (1 μμ) and 4-AP (100 μM) were routinely included to isolate optogenetically evoked monosynaptic thalamocortical or corticocortical EPSCs in M1. To test the effectiveness of thalamic and cortical driving of M1 neuronal firing (Fig. 4), TTX/4-AP were omitted from the recording solution, and a selective GABA_A_ receptor antagonist SR-95531 (GABAzine) was added to prevent the recruitment of GABAergic inhibitions. Neurons were visualized using a charge-coupled device camera (SciCam Pro, Scientifica, UK) and a SliceScope Pro 6000 system (Scientifica, UK) integrated with a BX51 upright microscope and motorized micromanipulators. Retrogradely labeled neurons in the layer V were targeted under a 60× water immersion objective lens (Olympus, Japan) for whole-cell patch clamp recording using a MultiClamp 700B amplifier (RRID:SCR_018455) and Digidata 1550B controlled by pClamp 11 software (Molecular Devices, San Jose, CA; RRID:SCR_011323). Data were sampled at 50 KHz. Borosilicate glass pipettes (outer diameter = 1.5 mm, inner diameter = 0.86 mm, length = 10 cm, item no. BF150-86-10, Sutter Instruments, Novato, CA) were pulled by micropipette puller (P1000, Sutter instrument, Novato, CA; RRID:SCR_021042) and used for patch clamp recording with a resistance of 3 to 6 megohm when filled with (i) Cs-methanesulfonate–based internal solution of 120 mM CH_3_O_3_SCs, 2.8 mM NaCl, 10 mM Hepes, 0.4 mM Na_4_-EGTA, 5 mM QX314-HBr, 5 mM phosphocreatine, 0.1 mM spermine, 4 mM ATP-Mg, and 0.4 mM GTP-Na (pH 7.3, 290 mOsm); or (ii) k-gluconate–based internal solution, containing 140 mM k-gluconate, 3.8 mM NaCl, 1 mM MgCl_2_, 10 mM Hepes, 0.1 mM Na_4_-EGTA, 2 mM ATP-Mg, and 0.1 mM GTP-Na (pH 7.3, 290 mOsm). Biocytin (0.2%) was added to the k-gluconate–based internal solution to fill retrobead-labeled PT neurons (~ 30 min), and the morphology of the filled neuron was revealed using Cy5-conjugated streptavidin. Spontaneous inhibitory postsynaptic currents from the motor thalamic neurons were conducted in the presence of glutamatergic receptor blockers (i.e., 50 μM D-APV and 20 μM DNQX) under whole-cell voltage-clamp mode (at −70 mV) using a high Cl^−^ internal solution, containing 135 mM CsCl, 3.6 mM NaCl, 1 mM MgCl_2_, 10 mM Hepes, 0.1 mM Na_4_-EGTA, 2 mM ATP-Mg, and 0.1 mM GTP-Na (pH 7.3, 290 mOsm). Optogenetic stimulation was delivered using a 478-nm light-emitting diode (LED; pE-300^Ultra^, CoolLED, UK; RRID:SCR_021972) through a 60× water immersion objective lens (Olympus), centering at the site of patched neurons with a field of illumination of ~450 μm in diameter. In chemogenetics studies, water-soluble CNO (10 μμ, HelloBio no. HB6149) was applied ex vivo to test hM4Di activation on SNr neuronal firing or sIPSCs in the VM thalamic neurons. Τhe effects of CNO on neuronal firing or sIPSCs were assessed 15 to 20 min after the start of CNO perfusion. Electrophysiology data were analyzed and quantified using Clampfit 11.1 (Molecular Devices, San Jose, CA; RRID:SCR_011323) and Minianalysis 6.0.3 (Synaptosoft, USA; RRID:SCR_002184). Peak amplitude of AMPARs-mediated EPSCs at −80 mV was quantified as a measure of synaptic strength. To calculate NMDA/AMPA ratio, amplitude of NMDA-mediated currents at +40 mV was measured at 50-ms post-optogenetic stimulation when AMPAR EPSCs largely decay to baseline level. Details of brain slice physiology and optogenetics can be found at Protocols.io (dx.doi.org/10.17504/protocols.io.eq2ly7m2rlx9/v1).

### Immunohistochemistry

Brain tissues after electrophysiology or NMDA incubation (1 hour) were fixed in 4% PFA in 0.1 M phosphate buffer overnight at 4°C and then were resected into 70- to 100-μm slices using a VT1000s vibratome (Leica Biosystems, Deer Park, IL; RRID:SCR_016495) to prepare brain sections for immunohistochemistry of tyrosine hydroxylase (TH) or the marker of neuronal nuclei (NeuN). In vGluT2 stereology and neuronal morphology studies, mice were perfused with ice-cold phosphate-buffered saline (PBS; pH = 7.4) for 5 min and subsequently with 4% PFA mice for 30 min. The brain was then extracted and saved in 4% PFA overnight at 4°C before being resected into 70-μm slices using a VT1000s vibratome (Leica Biosystems, Deer Park, IL; RRID:SCR_016495) for immunohistochemistry or morphology studies.

Brain slices were rinsed 3× with PBS before being incubated with 0.2% Triton X-100 and 2% normal donkey serum (Sigma-Aldrich) for 60 min at room temperature. Brain slices were then incubated with primary antibodies, including mouse anti-TH (1:2000; catalog no. MAB318, MilliporeSigma; RRID:AB_2201528), anti-NeuN (MAB377, MilliporeSigma, RRID:AB_2298772), or guinea pig anti-vGluT2 (catalog no. 135404, Synaptic Systems, RRID:AB_887884) for 48 hours at 4°C or overnight at room temperature. Sections were then rinsed 3× with PBS and incubated with secondary antibodies for 90 min, including donkey anti-mouse Alexa Fluor 488 (catalog no. 715-545-150; Jackson ImmunoResearch Labs, West Grove, PA; RRID:AB_2340846), donkey anti-mouse Alexa Fluor 594 (catalog no. 715-585-150; Jackson ImmunoResearch Labs, West Grove, PA; RRID:AB_2340854), or donkey anti-guinea pig Alexa Fluor 488 (catalog no. 706-545-148; Jackson ImmunoResearch Labs, West Grove, PA; RRID:AB_2340472), at room temperature before washing with PBS for three times. Brain sections were mounted with VECTASHIELD antifade mounting medium (catalog no. H-1000, Vector Laboratories, Newark, CA; RRID:AB_2336789) and were cover-slipped and sealed with nail polish. TH and vGluT2 immunoreactivity (ir) was imaged under 20× or 40× objective lens using an Olympus DP80 camera through an Olympus BX63F microscope or a confocal laser scanning microscope (A1R, Nikon, Japan; RRID:SCR_020317). TH-ir of control and 6-OHDA mice was quantified by normalizing the striatal TH-ir from the ipsilateral side to the value from the contralateral hemisphere, with a subtraction of background measured from the cerebral cortex. Details of immunohistochemical quantification of TH signals can be found at Protocols.io (dx.doi.org/10.17504/protocols.io.n2bvj85qngk5/v1).

### Animal behavior

To assess parkinsonian motor deficits, mice were subject to (i) open field locomotion test, where their locomotor activity and rotations were monitored for 10 min and quantified using Anymaze software (Stoelting, Wood Dale, IL); (ii) cylinder test using a 600-ml glass beaker to assess the spontaneous forelimbs use during weight-bearing touches which were recorded using a digital camcorder at 60 fps (Panasonic, HC-V180K) and analyzed off-line by a researcher blinded to treatments. Three weeks postsurgery, vehicle- or 6-OHDA–injected mice received subcutaneous saline- or water-soluble CNO (1 mg/kg body weight, catalog no. HB6149, HelloBio, Princeton, NJ) injections for chemogenetic studies. The injections were conducted at 7 a.m. and 7 p.m. each day for five consecutive days. Locomotion tests (10 min) were performed 30 min after subcutaneous injections on days 1, 3, and 5 to measure the behavioral effects. Following behavioral tests, animals were used to prepare brain slices for physiology studies. All physiology recordings were started ~3 hours after saline or CNO injections. Details of locomotion and cylinder tests can be found at Protocols.io (dx.doi.org/10.17504/protocols.io.e6nvwjxmdlmk/v1 and dx.doi.org/10.17504/protocols.io.eq2ly7m9qlx9/v1).

### Validation of mouse model of parkinsonism

To confirm a complete degeneration of nigrostriatal DA pathway in 6-OHDA mice, animals were subject to behavioral tests at 3 weeks postsurgery, before electrophysiology and anatomy experiments (*26*). Relative to controls, 6-OHDA mice showed a substantial reduction of locomotor activity in an open field arena and asymmetric forelimb use in cylinder test. Post hoc immunostaining of striatal TH was conducted to confirm that there was >80% loss of striatal TH-ir in the lesioned hemispheres of 6-OHDA mice. Mice that received 6-OHDA injection but showed partial striatal DA depletion (i.e., striatal TH loss was <80%) were excluded from further analyses. Procedures of TH quantification and behavioral validation of parkinsonism can be found on Protocols.io (dx.doi.org/10.17504/protocols.io.n2bvj85qngk5/v1, dx.doi.org/10.17504/protocols.io.e6nvwjxmdlmk/v1, and dx.doi.org/10.17504/protocols.io.eq2ly7m9qlx9/v1).

### Confocal imaging and digital image analysis

vGluT2-ir was collected from layers V under a 100× objective lens using a Nikon A1R confocal microscope. Z stack Images with a 0.15-μm interval were taken under the identical settings, including laser power, gain, pinhole size, and scanning speed between animals. The density of vGluT2-ir was estimated on the basis of the immunostaining signals between 5 and 8 μm below the surface of slices using the optical dissector method (*65*). To assess the density of neurons in layer V, NeuN-ir was collected using a 40× objective lens (1024 × 1024 pixels; *z* step = 1 μm, Nikon A1R) followed by stereology analysis. For spine density analysis and dendritic examination, segments of basal dendrites were tracked down from the cell bodies of retrogradely labeled PT neurons in M1 and imaged under an oil-immersion 100× objective using the confocal microscope (numerical aperture = 1.45; *x*/*y*, 1024 × 1024 pixels; *z* step = 0.5 μm, A1R, Nikon). The distance from the soma to the basal dendrites analyzed was 50 to 100 μm. eGFP- or biocytin-labeled segments of the apical dendrites were selected from layers I and II/III. To quantify spine density, only microscopic image data with ample eGFP expression and high signal-to-noise ratios were included. Spine density analysis was performed using Imaris software (version 9.3, Oxford, UK; RRID:SCR_007370). Briefly, two to three dendritic segments from the targeted layers measuring 20 to 30 μm in length were reconstructed three-dimensionally using Imaris’ filament tracer function from each confocal image. Spines were then manually traced, reconstructed, and quantified. For accurate three-dimensionally reconstruction, both the dendrite segments and spines were recomputed automatically based on the manual traces. Details of imaging process can be found at Protocols.io (dx.doi.org/10.17504/protocols.io.3byl4jmxzlo5/v1).

### Statistics

Statistics analysis was performed in GraphPad Prism 9 (GraphPad Software, San Diego, CA; RRID:SCR_002798). Nonparametric Mann-Whitney *U* (MWU) test, Wilcox signed rank (WSR) test, and Kruskal-Wallis test followed by Dunn’s multiple comparisons (as indicated in the text) were used to compare the medians of two or three groups to minimize the assumption of dataset normality. For experiments with nested design wherein more than one cell were sampled from each animal, a mixed effects model was used to compare group difference where the responses (e.g., EPSC amplitude) were modeled as a combination of a fixed effect (i.e., vehicle versus 6-OHDA injections or saline versus CNO injections) and random effects associated with each animal. Behavioral results in the Fig. 8 were analyzed using repeated measures two-way analysis of variance (ANOVA). Whenever applicable, post hoc Šídák’s multiple comparisons test was performed. Data were collected from three to eight mice per study. Individual animals were considered independent samples. All statistic tests were two tailed with *P* values < 0.05 (*), 0.01 (**), or 0.001 (***) as thresholds for statistical significance. Data are reported as median plus interquartile ranges (in square brackets).

## References

[1] R. L. Albin, A. B. Young, J. B. Penney, The functional anatomy of basal ganglia disorders. Trends Neurosci. 12, 366–375 (1989). 2479133 10.1016/0166-2236(89)90074-x

[2] A. Galvan, T. Wichmann, Pathophysiology of parkinsonism. Clin. Neurophysiol. 119, 1459–1474 (2008). 18467168 10.1016/j.clinph.2008.03.017 PMC2467461

[3] M. M. McGregor, A. B. Nelson, Circuit mechanisms of Parkinson’s disease. Neuron 101, 1042–1056 (2019). 30897356 10.1016/j.neuron.2019.03.004

[4] K. C. Nakamura, A. Sharott, P. J. Magill, Temporal coupling with cortex distinguishes spontaneous neuronal activities in identified basal ganglia-recipient and cerebellar-recipient zones of the motor thalamus. Cereb. Cortex 24, 81–97 (2014). 23042738 10.1093/cercor/bhs287 PMC3862266

[5] E. Kuramoto, T. Furuta, K. C. Nakamura, T. Unzai, H. Hioki, T. Kaneko, Two types of thalamocortical projections from the motor thalamic nuclei of the rat: A single neuron-tracing study using viral vectors. Cereb. Cortex 19, 2065–2077 (2009). 19174446 10.1093/cercor/bhn231

[6] Á. L. Bodor, K. Giber, Z. Rovó, I. Ulbert, L. Acsády, Structural correlates of efficient GABAergic transmission in the basal ganglia–thalamus pathway. J. Neurosci. 28, 3090–3102 (2008). 18354012 10.1523/JNEUROSCI.5266-07.2008 PMC2670451

[7] L. E. McElvain, Y. Chen, J. D. Moore, G. S. Brigidi, B. L. Bloodgood, B. K. Lim, R. M. Costa, D. Kleinfeld, Specific populations of basal ganglia output neurons target distinct brain stem areas while collateralizing throughout the diencephalon. Neuron 109, 1721–1738.e4 (2021). 33823137 10.1016/j.neuron.2021.03.017 PMC8169061

[8] B. M. Hooks, T. Mao, D. A. Gutnisky, N. Yamawaki, K. Svoboda, G. M. G. Shepherd, Organization of cortical and thalamic input to pyramidal neurons in mouse motor cortex. J. Neurosci. 33, 748–760 (2013). 23303952 10.1523/JNEUROSCI.4338-12.2013 PMC3710148

[9] M. Gaidica, A. Hurst, C. Cyr, D. K. Leventhal, Distinct populations of motor thalamic neurons encode action initiation, action selection, and movement vigor. J. Neurosci. 38, 6563–6573 (2018). 29934350 10.1523/JNEUROSCI.0463-18.2018 PMC6052244

[10] C. Bosch-Bouju, B. I. Hyland, L. C. Parr-Brownlie, Motor thalamus integration of cortical, cerebellar and basal ganglia information: Implications for normal and parkinsonian conditions. Front. Comput. Neurosci. 7, 163 (2013). 24273509 10.3389/fncom.2013.00163 PMC3822295

[11] N. Takahashi, S. Moberg, T. A. Zolnik, J. Catanese, R. N. S. Sachdev, M. E. Larkum, D. Jaeger, Thalamic input to motor cortex facilitates goal-directed action initiation. Curr. Biol. 31, 4148–4155.e4 (2021). 34302741 10.1016/j.cub.2021.06.089 PMC8478854

[12] F. Aeed, N. Cermak, J. Schiller, Y. Schiller, Intrinsic disruption of the M1 cortical network in a mouse model of Parkinson’s disease. Mov. Disord. 36, 1565–1577 (2021). 33606292 10.1002/mds.28538

[13] B. I. Hyland, S. Seeger-Armbruster, R. A. Smither, L. C. Parr-Brownlie, Altered recruitment of motor cortex neuronal activity during the grasping phase of skilled reaching in a chronic rat model of unilateral Parkinsonism. J. Neurosci. 39, 9660–9672 (2019). 31641050 10.1523/JNEUROSCI.0720-19.2019 PMC6880456

[14] B. Pasquereau, M. R. DeLong, R. S. Turner, Primary motor cortex of the parkinsonian monkey: Altered encoding of active movement. Brain 139, 127–143 (2016). 26490335 10.1093/brain/awv312 PMC4794619

[15] C. Bosch-Bouju, R. A. Smither, B. I. Hyland, L. C. Parr-Brownlie, Reduced reach-related modulation of motor thalamus neural activity in a rat model of Parkinson’s disease. J. Neurosci. 34, 15836–15850 (2014). 25429126 10.1523/JNEUROSCI.0893-14.2014 PMC6608476

[16] E. Brazhnik, A. J. McCoy, N. Novikov, C. E. Hatch, J. R. Walters, Ventral medial thalamic nucleus promotes synchronization of increased high beta oscillatory activity in the basal ganglia–thalamocortical network of the hemiparkinsonian rat. J. Neurosci. 36, 4196–4208 (2016). 27076419 10.1523/JNEUROSCI.3582-15.2016 PMC4829645

[17] B. A. Sauerbrei, J.-Z. Guo, J. D. Cohen, M. Mischiati, W. Guo, M. Kabra, N. Verma, B. Mensh, K. Branson, A. W. Hantman, Cortical pattern generation during dexterous movement is input-driven. Nature 577, 386–391 (2020). 31875851 10.1038/s41586-019-1869-9 PMC6962553

[18] K. V. Shenoy, M. Sahani, M. M. Churchland, Cortical control of arm movements: A dynamical systems perspective. Annu. Rev. Neurosci. 36, 337–359 (2012). 10.1146/annurev-neuro-062111-150509 23725001

[19] A. P. Georgopoulos, A. F. Carpenter, Coding of movements in the motor cortex. Curr. Opin. Neurobiol. 33, 34–39 (2015). 25646932 10.1016/j.conb.2015.01.012

[20] M. R. DeLong, Primate models of movement disorders of basal ganglia origin. Trends Neurosci. 13, 281–285 (1990). 1695404 10.1016/0166-2236(90)90110-v

[21] B. Pasquereau, R. S. Turner, Primary motor cortex of the parkinsonian monkey: Differential effects on the spontaneous activity of pyramidal tract-type neurons. Cereb. Cortex 21, 1362–1378 (2011). 21045003 10.1093/cercor/bhq217 PMC3097989

[22] J. A. Goldberg, T. Boraud, S. Maraton, S. N. Haber, E. Vaadia, H. Bergman, Enhanced synchrony among primary motor cortex neurons in the 1-methyl-4-phenyl-1,2,3,6-tetrahydropyridine primate model of Parkinson’s disease. J. Neurosci. 22, 4639–4653 (2002). 12040070 10.1523/JNEUROSCI.22-11-04639.2002 PMC6758785

[23] L. Guo, H. Xiong, J.-I. Kim, Y.-W. Wu, R. R. Lalchandani, Y. Cui, Y. Shu, T. Xu, J. B. Ding, Dynamic rewiring of neural circuits in the motor cortex in mouse models of Parkinson’s disease. Nat. Neurosci. 18, 1299–1309 (2015). 26237365 10.1038/nn.4082 PMC4551606

[24] R. M. Villalba, J. A. Behnke, J.-F. Pare, Y. Smith, Comparative ultrastructural analysis of thalamocortical innervation of the primary motor cortex and supplementary motor area in control and MPTP-treated Parkinsonian monkeys. Cereb. Cortex 31, 3408–3425 (2021). 33676368 10.1093/cercor/bhab020 PMC8599722

[25] O. K. Swanson, R. Semaan, A. Maffei, Reduced dopamine signaling impacts pyramidal neuron excitability in mouse motor cortex. Eneuro 8, ENEURO.0548-19.2021 (2021). 10.1523/ENEURO.0548-19.2021 PMC8525657 34556558

[26] L. Chen, S. Daniels, Y. Kim, H.-Y. Chu, Cell type-specific decrease of the intrinsic excitability of motor cortical pyramidal neurons in Parkinsonism. J. Neurosci. 41, 5553–5565 (2021). 34006589 10.1523/JNEUROSCI.2694-20.2021 PMC8221604

[27] A. R. Brown, B. Hu, M. C. Antle, G. C. Teskey, Neocortical movement representations are reduced and reorganized following bilateral intrastriatal 6-hydroxydopamine infusion and dopamine type-2 receptor antagonism. Exp. Neurol. 220, 162–170 (2009). 19703443 10.1016/j.expneurol.2009.08.015

[28] R. Viaro, M. Morari, G. Franchi, Progressive motor cortex functional reorganization following 6-hydroxydopamine lesioning in rats. J. Neurosci. 31, 4544–4554 (2011). 21430155 10.1523/JNEUROSCI.5394-10.2011 PMC6622898

[29] E. K. Plowman, N. J. Thomas, J. A. Kleim, Striatal dopamine depletion induces forelimb motor impairments and disrupts forelimb movement representations within the motor cortex. J. Parkinsons Dis. 1, 93–100 (2011). 23939260 10.3233/JPD-2011-11017 PMC7236202

[30] J. S. Biane, Y. Takashima, M. Scanziani, J. M. Conner, M. H. Tuszynski, Thalamocortical projections onto behaviorally relevant neurons exhibit plasticity during adult motor learning. Neuron 89, 1173–1179 (2016). 26948893 10.1016/j.neuron.2016.02.001 PMC4795975

[31] L. Petreanu, D. Huber, A. Sobczyk, K. Svoboda, Channelrhodopsin-2–assisted circuit mapping of long-range callosal projections. Nat. Neurosci. 10, 663–668 (2007). 17435752 10.1038/nn1891

[32] N. Yamawaki, G. M. G. Shepherd, Synaptic circuit organization of motor corticothalamic neurons. J. Neurosci. 35, 2293–2307 (2015). 25653383 10.1523/JNEUROSCI.4023-14.2015 PMC4315846

[33] A. Rios, S. Soma, J. Yoshida, S. Nonomura, M. Kawabata, Y. Sakai, Y. Isomura, Differential changes in the lateralized activity of identified projection neurons of motor cortex in hemiparkinsonian rats. Eneuro 6, ENEURO.0110-19.2019 (2019). 10.1523/ENEURO.0110-19.2019 PMC6620387 31235466

[34] H.-Y. Chu, E. L. McIver, R. F. Kovaleski, J. F. Atherton, M. D. Bevan, Loss of hyperdirect pathway cortico-subthalamic inputs following degeneration of midbrain dopamine neurons. Neuron 95, 1306–1318.e5 (2017). 28910619 10.1016/j.neuron.2017.08.038 PMC5679443

[35] J. Ding, J. D. Peterson, D. J. Surmeier, Corticostriatal and thalamostriatal synapses have distinctive properties. J. Neurosci. 28, 6483–6492 (2008). 18562619 10.1523/JNEUROSCI.0435-08.2008 PMC3461269

[36] R. M. Bruno, B. Sakmann, Cortex is driven by weak but synchronously active thalamocortical synapses. Science 312, 1622–1627 (2006). 16778049 10.1126/science.1124593

[37] A. Oswal, P. Brown, V. Litvak, Synchronized neural oscillations and the pathophysiology of Parkinsonʼs disease. Curr. Opin. Neurol. 26, 662–670 (2013). 24150222 10.1097/WCO.0000000000000034

[38] K. C. Nakamura, A. Sharott, T. Tanaka, P. J. Magill, Input zone-selective dysrhythmia in motor thalamus after dopamine depletion. J. Neurosci. 41, 10382–10404 (2021). 34753740 10.1523/JNEUROSCI.1753-21.2021 PMC8672689

[39] O. H. Miller, A. Bruns, I. B. Ammar, T. Mueggler, B. J. Hall, Synaptic regulation of a thalamocortical circuit controls depression-related behavior. Cell Rep. 20, 1867–1880 (2017). 28834750 10.1016/j.celrep.2017.08.002

[40] H. Wang, G. G. Stradtman, X.-J. Wang, W.-J. Gao, A specialized NMDA receptor function in layer 5 recurrent microcircuitry of the adult rat prefrontal cortex. Proc. Natl. Acad. Sci. U.S.A. 105, 16791–16796 (2008). 18922773 10.1073/pnas.0804318105 PMC2575498

[41] E. L. McIver, J. F. Atherton, H.-Y. Chu, K. E. Cosgrove, J. Kondapalli, D. Wokosin, D. J. Surmeier, M. D. Bevan, Maladaptive downregulation of autonomous subthalamic nucleus activity following the loss of midbrain dopamine neurons. Cell Rep. 28, 992–1002.e4 (2019). 31340159 10.1016/j.celrep.2019.06.076 PMC6699776

[42] J. F. Atherton, E. L. McIver, M. R. Mullen, D. L. Wokosin, D. J. Surmeier, M. D. Bevan, Early dysfunction and progressive degeneration of the subthalamic nucleus in mouse models of Huntington’s disease. eLife 5, e21616 (2016). 27995895 10.7554/eLife.21616 PMC5199195

[43] R. Levy, J. O. Dostrovsky, A. E. Lang, E. Sime, W. D. Hutchison, A. M. Lozano, Effects of apomorphine on subthalamic nucleus and globus pallidus internus neurons in patients with Parkinson’s disease. J. Neurophysiol. 86, 249–260 (2001). 11431506 10.1152/jn.2001.86.1.249

[44] M. Filion, L. Tremblay, P. J. Bédard, Effects of dopamine agonists on the spontaneous activity of globus pallidus neurons in monkeys with MPTP-induced parkinsonism. Brain Res. 547, 145–149 (1991). 1677608

[45] M. Merello, J. Balej, M. Delfino, A. Cammarota, O. Betti, R. Leiguarda, Apomorphine induces changes in GPi spontaneous outflow in patients with parkinson’s disease. Mov. Disord. 14, 45–49 (1999). 9918343 10.1002/1531-8257(199901)14:1<45::aid-mds1009>3.0.co;2-f

[46] J. A. Obeso, M. Jahanshahi, L. Alvarez, R. Macias, I. Pedroso, L. Wilkinson, N. Pavon, B. Day, S. Pinto, M. C. Rodríguez-Oroz, J. Tejeiro, J. Artieda, P. Talelli, O. Swayne, R. Rodríguez, K. Bhatia, M. Rodriguez-Diaz, G. Lopez, J. Guridi, J. C. Rothwell, What can man do without basal ganglia motor output? The effect of combined unilateral subthalamotomy and pallidotomy in a patient with Parkinson’s disease. Exp. Neurol. 220, 283–292 (2009). 19744484 10.1016/j.expneurol.2009.08.030

[47] T. Wichmann, Changing views of the pathophysiology of Parkinsonism. Mov. Disord. 34, 1130–1143 (2019). 31216379 10.1002/mds.27741

[48] J. Kim, Y. Kim, R. Nakajima, A. Shin, M. Jeong, A. H. Park, Y. Jeong, S. Jo, S. Yang, H. Park, S.-H. Cho, K.-H. Cho, I. Shim, J. H. Chung, S.-B. Paik, G. J. Augustine, D. Kim, Inhibitory basal ganglia inputs induce excitatory motor signals in the thalamus. Neuron 95, 1181–1196.e8 (2017). 28858620 10.1016/j.neuron.2017.08.028

[49] S. Seeger-Armbruster, C. Bosch-Bouju, S. T. C. Little, R. A. Smither, S. M. Hughes, B. I. Hyland, L. C. Parr-Brownlie, Patterned, but not tonic, optogenetic stimulation in motor thalamus improves reaching in acute drug-induced Parkinsonian rats. J. Neurosci. 35, 1211–1216 (2015). 25609635 10.1523/JNEUROSCI.3277-14.2015 PMC6605530

[50] T. K. Roseberry, A. M. Lee, A. L. Lalive, L. Wilbrecht, A. Bonci, A. C. Kreitzer, Cell-type-specific control of brainstem locomotor circuits by basal ganglia. Cell 164, 526–537 (2016). 26824660 10.1016/j.cell.2015.12.037 PMC4733247

[51] L. Wang, J. M. Conner, J. Rickert, M. H. Tuszynski, Structural plasticity within highly specific neuronal populations identifies a unique parcellation of motor learning in the adult brain. Proc. Natl. Acad. Sci. U.S.A. 108, 2545–2550 (2011). 21257908 10.1073/pnas.1014335108 PMC3038698

[52] R. Hasegawa, T. Ebina, Y. R. Tanaka, K. Kobayashi, M. Matsuzaki, Structural dynamics and stability of corticocortical and thalamocortical axon terminals during motor learning. PLOS ONE 15, e0234930 (2020). 32559228 10.1371/journal.pone.0234930 PMC7304593

[53] B. A. Suter, M. Migliore, G. M. G. Shepherd, Intrinsic electrophysiology of mouse corticospinal neurons: A class-specific triad of spike-related properties. Cereb. Cortex 23, 1965–1977 (2013). 22761308 10.1093/cercor/bhs184 PMC3698370

[54] G. M. G. Shepherd, Corticostriatal connectivity and its role in disease. Nat. Rev. Neurosci. 14, 278–291 (2013). 23511908 10.1038/nrn3469 PMC4096337

[55] H. Peng, P. Xie, L. Liu, X. Kuang, Y. Wang, L. Qu, H. Gong, S. Jiang, A. Li, Z. Ruan, L. Ding, Z. Yao, C. Chen, M. Chen, T. L. Daigle, R. Dalley, Z. Ding, Y. Duan, A. Feiner, P. He, C. Hill, K. E. Hirokawa, G. Hong, L. Huang, S. Kebede, H.-C. Kuo, R. Larsen, P. Lesnar, L. Li, Q. Li, X. Li, Y. Li, Y. Li, A. Liu, D. Lu, S. Mok, L. Ng, T. N. Nguyen, Q. Ouyang, J. Pan, E. Shen, Y. Song, S. M. Sunkin, B. Tasic, M. B. Veldman, W. Wakeman, W. Wan, P. Wang, Q. Wang, T. Wang, Y. Wang, F. Xiong, W. Xiong, W. Xu, M. Ye, L. Yin, Y. Yu, J. Yuan, J. Yuan, Z. Yun, S. Zeng, S. Zhang, S. Zhao, Z. Zhao, Z. Zhou, Z. J. Huang, L. Esposito, M. J. Hawrylycz, S. A. Sorensen, X. W. Yang, Y. Zheng, Z. Gu, W. Xie, C. Koch, Q. Luo, J. A. Harris, Y. Wang, H. Zeng, Morphological diversity of single neurons in molecularly defined cell types. Nature 598, 174–181 (2021). 34616072 10.1038/s41586-021-03941-1 PMC8494643

[56] M. Lafourcade, M.-S. H. van der Goes, D. Vardalaki, N. J. Brown, J. Voigts, D. H. Yun, M. E. Kim, T. Ku, M. T. Harnett, Differential dendritic integration of long-range inputs in association cortex via subcellular changes in synaptic AMPA-to-NMDA receptor ratio. Neuron 110, 1532–1546.e4 (2022). 35180389 10.1016/j.neuron.2022.01.025 PMC9081173

[57] E. M. Lewis, H. E. Spence, N. Akella, A. Buonanno, Pathway-specific contribution of parvalbumin interneuron NMDARs to synaptic currents and thalamocortical feedforward inhibition. Mol. Psychiatry 27, 5124–5134 (2022). 36075962 10.1038/s41380-022-01747-9 PMC9763122

[58] K. Shima, J. Tanji, Involvement of NMDA and Non-NMDA receptors in the neuronal responses of the primary motor cortex to input from the supplementary motor area and somatosensory cortex: Studies of task-performing monkeys. Jpn. J. Physiol. 48, 275–290 (1998). 9757144 10.2170/jjphysiol.48.275

[59] R. Meller, S. J. Thompson, T. A. Lusardi, A. N. Ordonez, M. D. Ashley, V. Jessick, W. Wang, D. J. Torrey, D. C. Henshall, P. R. Gafken, J. A. Saugstad, Z.-G. Xiong, R. P. Simon, Ubiquitin proteasome-mediated synaptic reorganization: A novel mechanism underlying rapid ischemic tolerance. J. Neurosci. 28, 50–59 (2008). 18171922 10.1523/JNEUROSCI.3474-07.2008 PMC2946223

[60] M. J. Hasbani, M. L. Schlief, D. A. Fisher, M. P. Goldberg, Dendritic spines lost during glutamate receptor activation reemerge at original sites of synaptic contact. J. Neurosci. 21, 2393–2403 (2001). 11264313 10.1523/JNEUROSCI.21-07-02393.2001 PMC6762381

[61] H. J. Kim, J. J. Waataja, S. A. Thayer, Cannabinoids inhibit network-driven synapse loss between hippocampal neurons in culture. J. Pharmacol. Exp. Ther. 325, 850–858 (2008). 18310474 10.1124/jpet.107.131607 PMC2398764

[62] S. Graber, S. Maiti, S. Halpain, Cathepsin B-like proteolysis and MARCKS degradation in sub-lethal NMDA-induced collapse of dendritic spines. Neuropharmacology 47, 706–713 (2004). 15458842 10.1016/j.neuropharm.2004.08.004

[63] J. Cousineau, V. Plateau, J. Baufreton, M. L. Bon-Jégo, Dopaminergic modulation of primary motor cortex: From cellular and synaptic mechanisms underlying motor learning to cognitive symptoms in Parkinson’s disease. Neurobiol. Dis. 167, 105674 (2022). 35245676 10.1016/j.nbd.2022.105674

[64] J. A. Hosp, H. E. Nolan, A. R. Luft, Topography and collateralization of dopaminergic projections to primary motor cortex in rats. Exp. Brain Res. 233, 1365–1375 (2015). 25633321 10.1007/s00221-015-4211-2

[65] M. J. West, Stereological methods for estimating the total number of neurons and synapses: Issues of precision and bias. Trends Neurosci. 22, 51–61 (1999). 10092043 10.1016/s0166-2236(98)01362-9

