# Supplementary material for: Reduced thalamic excitation to motor cortical pyramidal tract neurons in parkinsonism
Source: Sci Adv. 2023 Aug 23;9(34):eadg3038. doi: 10.1126/sciadv.adg3038 (PMC10446482; doi:10.1126/sciadv.adg3038)
Supplement: Supplementary file 1 — Figs. S1 to S8 [file sciadv.adg3038_sm.pdf]

Science Advances
AAAS

Supplementary Materials for

Reduced thalamic excitation to motor cortical pyramidal tract neurons in parkinsonism

Liqiang Chen *et al.*

Corresponding author: Hong-Yuan Chu, hongyuan.chu@vai.org

Sci. Adv. **9**, eadg3038 (2023)
DOI: 10.1126/sciadv.adg3038

This PDF file includes:

Figs. S1 to S8

Supplementary Figures

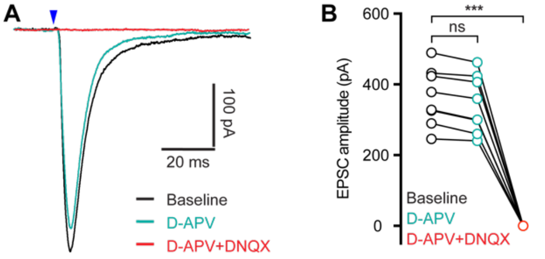

Figure S1 Optogenetic stimulation evokes glutamatergic thalamo-PT transmission. A) Representative traces show that thalamo-PT EPSCs at –80 mV were inhibited by D-APV (50 µM, NMDA receptor antagonist) and 6,7-dinitroquinoxaline-2,3-dione (DNQX, 20 µM, AMPA receptor antagonist). **B**) Summarized results confirmed a complete inhibition of thalamo-PT EPSCs after blockade glutamatergic receptors. Baseline vs D-APV, *p* > 0.99; baseline vs D-APV+DNQX, *p* = 0.0005. n = 8 neurons/3 mice, Kruskal-Wallist test.

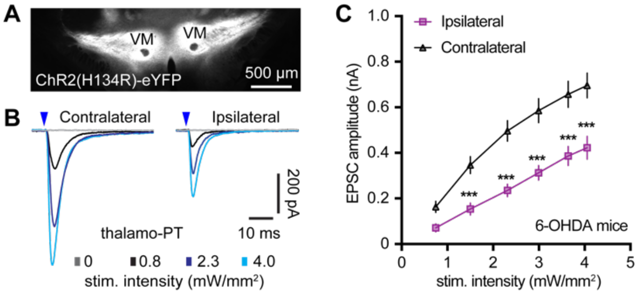

Figure S2 DA depletion does not decrease contralateral thalamo-PT transmission. A) Representative image showing ChR2 injection site in the mTh of a 6-OHDA mouse. **B)** Representative traces of EPSCs recorded from ipsilateral and contralateral hemispheres. **C)** Summarized data showing the reduced amplitude of EPSCs from the ipsilateral hemisphere relative to those from the contralateral hemisphere. *P* < 0.0001 between groups, mixed effects model followed by Sidak's tests.

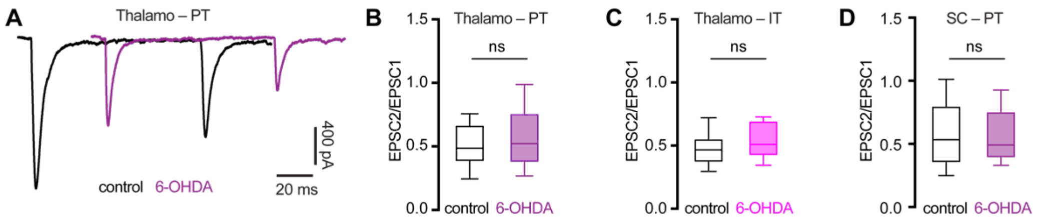

Figure S3. SNc DA degeneration does not affect the initial release probability of thalamic or sensory cortical inputs to M1 pyramidal neurons. A) Representative traces of EPSCs at the thalamo-PT synapses in response to paired pulses stimulation from controls and 6-OHDA mice. **B-D**) Box plots showing no change in ratio of EPSC2/EPSC1 at thalamo-PT (B, control = 0.49 [0.39–0.66], n= 62 neurons/7 mice; 6-OHDA = 0.52 [0.38–0.75], n = 73 neurons/8 mice; $p = 0.1$, MWU); C, thalamo-IT (control = 0.47 [0.37–0.55]), 6-OHDA = 0.51 [0.42–0.7], n = 26 neurons/3 mice; $p = 0.19$, MWU); and D, SC-PT synapses (control = 0.53 [0.35 –0.8], 6-OHDA = 0.49 [0.39–0.76], n= 20 neurons/3 mice; $p = 0.84$, MWU).

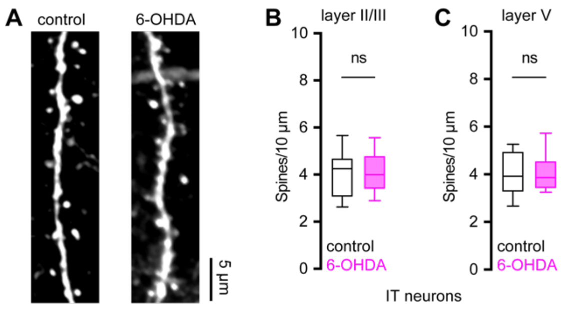

Figure S4 SNc DA degeneration does not affect spine density of IT neurons. A) Representative confocal images of eGFP-labeled dendrites of IT neurons in the layer V of M1. **B-C**) Box plots showing unaltered densities of IT neuronal spines in the layer II/III (B) and layer V (C) between control and 6-OHDA mice (layer II/III: control = 4.2 [3.1–4.7] spines/10μm, 6-OHDA = 4.0 [3.4–4.8] spines/10μm; layer V: control = 3.9 [3.3–5.0] spines/10μm, 6-OHDA = 3.9 [3.4–4.6] spines/10μm; n = 24 segments/3 mice, p > 0.7, MWU).

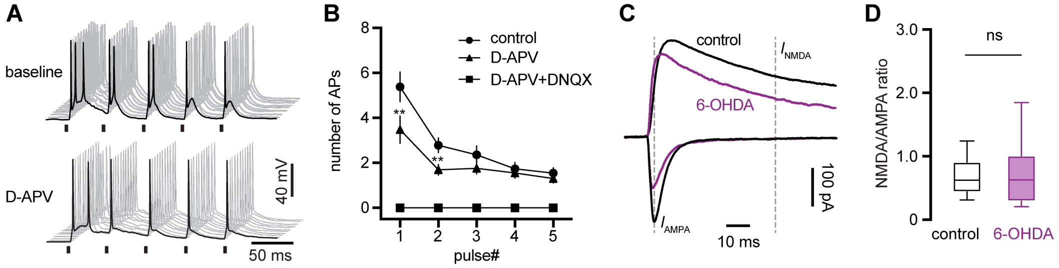

Figure S5. **NMDARs and AMPARs mediate thalamic driving of PT neuronal firing in control mice. A**) Representative traces of AP firing of M1 PT neurons in response to 20 Hz optogenetic stimulation prior to and after D-APV application. **B**) Summarized results show that NMDARs blockade by D-APV decreased the number of thalamus-driven APs of PT neurons. **, *p*< 0.01, mixed effects model followed by Sidak's tests; 13 neurons/3 mice. **C**) Representative traces of optogenetically-evoked thalamic EPSCs recorded at –80 mV and +40 mV in PT neurons from controls and 6-OHDA mice. Dashed lines indicate where AMPARs- and NMDARs-mediated responses were measured to quantify NMDA/APMA ratio. **D**) Summarized results showing no change in the NMDA/AMPA ratio between groups (control = 0.62 [0.44–0.91], n = 34 neurons/4 mice; 6-OHDA = 0.63 [0.29–1.0], n = 38 neurons/5 mice; *p* = 0.85, MWU).

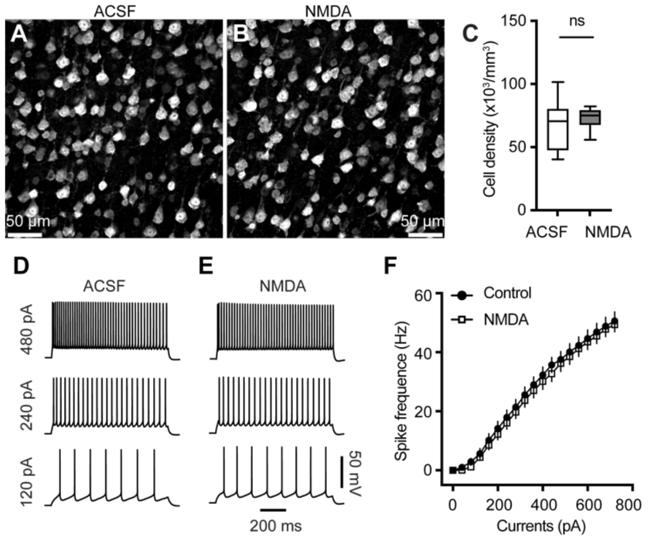

Figure S6. NMDARs stimulation did not induce cortical neurodegeneration or impair cellular excitability. A-C) The density of NeuN-positive ($NeuN^+$) neurons in layer V in brain slices prepared from control mice was not affected by *ex vivo* NMDA incubation (25 µM for 1 hour). A-B, Representative confocal images; C, Summarized results ($NeuN^+$ cell density, controls = 70 [48–80] $x10^3/mm^3$, 6-OHDA = 75 [68–79] $x10^3/mm^3$, *p* = 0.5, MWU test). **D-F**) *Ex vivo* NMDARs stimulation did not affect cellular excitability of PT neurons. **D-E**) Representative traces of action potentials of PT neurons from slices treated with ACSF or ACSF containing NMDA (25 µM). **F**) Summarized data. N = 21-24 neurons/3 mice for each group; Mixed effects model, *p* > 0.05.

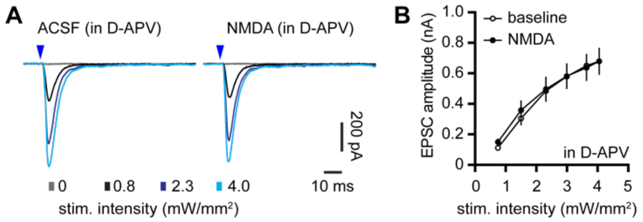

Figure S7. The effect of exogenous NMDA to thalamo-PT transmission can be abolished by D-APV. A) Representative traces of optogenetically-evoked thalamo-PT oEPSCs from slices treated with ASCF with D-APV (50 μM, left), and from slices treated with ACSF with D-PAV (50 μM) plus NMDA (25 μM, right). **B**) Summarized data showing exogenous NMDA did not alter the amplitude of thalamo-PT EPSCs in the presence of D-APV. N = 19 neurons/3 mice. Mixed effects model, $p > 0.05$.

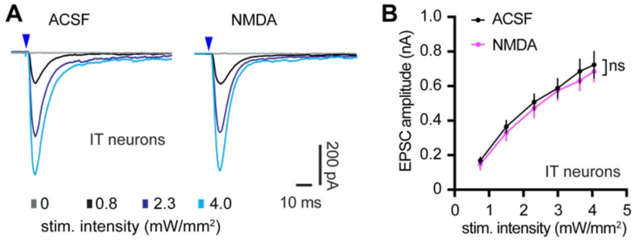

Figure S8 Thalamo-IT EPSCs in controls were not affected by *ex vivo* NMDA incubation. A) Representative traces of EPSCs from slices incubated with ACSF or NMDA-containing ACSF (25 μM for 1 hour. B) Group data confirm that NMDA incubation did not alter the amplitude of thalamo-IT EPSCs. 21-22 neurons/3 mice.
